# Dual-targeted microbubbles for atherosclerosis therapy: Inducing M1 macrophage apoptosis by inhibiting telomerase activity

**DOI:** 10.1016/j.mtbio.2025.101675

**Published:** 2025-03-20

**Authors:** Wei Zeng, Zhengan Huang, Yalan Huang, Kaifen Xiong, Yuanyuan Sheng, Xiaoxuan Lin, Xiaofang Zhong, Jiayu Ye, Yanbin Guo, Gulzira Arkin, Jinfeng Xu, Hongwen Fei, Yingying Liu

**Affiliations:** a Shenzhen Medical Ultrasound Engineering Center, Department of Ultrasonography, Shenzhen People's Hospital, Second Clinical Medical College of Jinan University, Shenzhen, 518020, China; b Guangdong Cardiovascular Institute, Guangdong Provincial People's Hospital (Guangdong Academy of Medical Sciences), Southern Medical University, Guangzhou, 519041, China; c Post-doctoral Scientific Research Station of Basic Medicine, Jinan University, Guangzhou, 510632, China; d Department of Dermatology, Shenzhen People's Hospital, Second Clinical Medical College of Jinan University, Shenzhen, 518020, China; e Department of Dermatology, Xiangya Hospital, Central South University, Changsha, 410000, China

**Keywords:** Magnetic microbubbles, Targeted delivery, Telomerase inhibitor, M1 macrophages, Atherosclerosis

## Abstract

The progression of atherosclerosis (AS) is closely associated with M1 macrophages. Although the activation of macrophage telomerase during plaque formation has been documented, targeted modulation strategies remain challenging. In this study, we developed a dual-target microbubble-delivery system (Ab-MMB_1532_) encapsulating BIBR1532, a telomerase inhibitor, for the targeted therapy of AS. This system exhibited remarkable targeting capabilities towards M1 macrophages, with its targeting advantage notably accentuated under high shear forces. Mechanistically, Ab-MMB_1532_ inhibited telomerase activity by downregulating telomerase reverse transcriptase (TERT) expression, subsequently inducing caspase-3-mediated apoptosis. Integrated multi-omics profiling revealed that the inhibition of the NF-κB pathway served as the central regulatory hub. *In vivo* studies further confirmed that Ab-MMB_1532_ effectively targets and accumulates within AS lesions, promoting M1 macrophage apoptosis through the inhibition of the TERT/NF-κB signaling axis, and significantly reducing plaque burden (25.4 % reduction vs. controls, *p* < 0.001). In summary, our findings suggest a novel approach for telomerase-targeted therapy in AS.

## Highlights

• Dual-target microbubble drug-delivery system for AS is developed.
• Targeting toward M1 macrophages is notably accentuated under high shear forces.
• Ab-MMB_1532_ downregulates telomerase reverse transcriptase expression.
• *In vivo* studies confirms that Ab-MMB_1532_ targets and anti-AS performance.

## Graphical abstract

**Figure:**
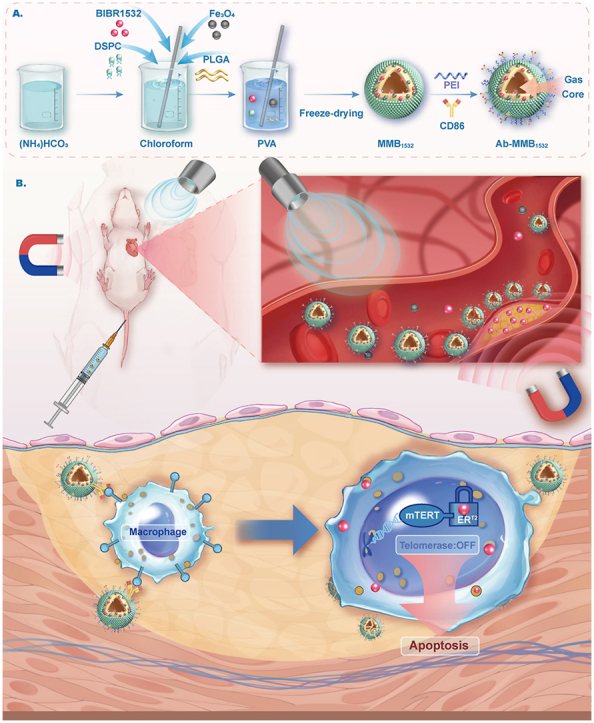

## 1 Introduction

Atherosclerosis (AS), a chronic inflammatory artery disease characterized by the accumulation of lipid and fibrous-element deposition underneath the inner wall of vessels, is a major cause of cardiovascular death, accounting for an estimated 17.9 million lives annually [1,2]. The growth of AS lesions or rupture of ‘vulnerable’ plaques can lead to acute myocardial infarction, stroke, and sudden cardiac death [3,4]. In such cases, early diagnosis and intervention of AS are vital for mitigating disease progression and reducing mortality. Currently, routine clinical treatments for AS include lowering lipid levels, controlling hypertension, antiplatelet therapy, and preventing blood-clot formation. However, the incidence and mortality of AS remain significantly high. Moreover, severe AS may require percutaneous and surgical management, which depends on expensive and invasive, albeit often effective, technology [5]. Therefore, novel therapeutic strategies against AS are urgently required.

Macrophages are key mediators of inflammatory and metabolic signals in atherosclerotic plaques [6]. The fate of macrophages depends on the inflammatory microenvironment within the plaque, which drives polarization towards either the classically activated pro-inflammatory M1 response or alternatively triggered M2 immune response [7]. M1 macrophages primarily promote the development of AS through inflammatory pathways, including inflammation-induced responses and positive feedback mechanisms mediated by the secretion of pro-inflammatory cytokines such as TNF-α, IL-1β, and IL-6 [8,9]. CD86, also known as B7.2, is a surface molecule that is predominantly expressed on antigen-presenting cells, including macrophages [10]. Its expression is closely associated with the pro-inflammatory phenotype of M1 macrophages and serves as a widely recognized marker of M1 macrophage activation [11], playing a crucial role in promoting the progression of AS plaques [12].

Telomerase is a critical factor in the regulation of tissue renewal and is involved in AS [13]. Telomerase is a ribonucleoprotein complex comprising several components, including telomerase reverse transcriptase (TERT), a telomerase RNA component that serves as a template for adding telomere repeats [14]. Evidence suggests that macrophage infiltration during AS progression can facilitate telomerase reactivation, resulting in a marked upregulation of TERT expression [15,16]. More importantly, telomerase activation has been shown to establish a positive feedback loop with the NF-κB signaling pathway, amplifying the expression of pro-inflammatory mediators, including M1 macrophage-associated markers such as MCP-1 and TNF-α [16]. This relationship exacerbates M1 macrophage-mediated inflammatory responses and accelerates AS progression. Therefore, targeting and inhibiting telomerase activity in M1 macrophages within atherosclerotic plaques may constitute a promising therapeutic approach. Considering that AS predominantly develops in large and medium arteries characterized by high blood-flow velocity and elevated shear stress [17], the effective and selective targeting of M1 macrophages within these regions has emerged as both a central challenge and the primary focus of this study.

Nanotechnology is particularly advantageous in addressing these challenges [18]. Recent advances have shown the potential of anti-AS drug-delivery systems based on ultrasound microbubbles (MB) [[19], [20], [21], [22]]. However, considerable challenges remain in terms of the targeting efficiency and controllable drug release at lesion sites caused by the shear forces of blood flow [23,24]. Fortunately, emerging magnetic ultrasound microbubble (MMB) navigation technology can alter the axial-distribution characteristics of MBs within blood vessels. Under the influence of an external magnetic field, the MMB can overcome shear forces, pushing it closer to the vessel wall, thereby increasing the opportunities for contact with target sites in the region of interest [[25], [26], [27]].

**Fig. 1:**
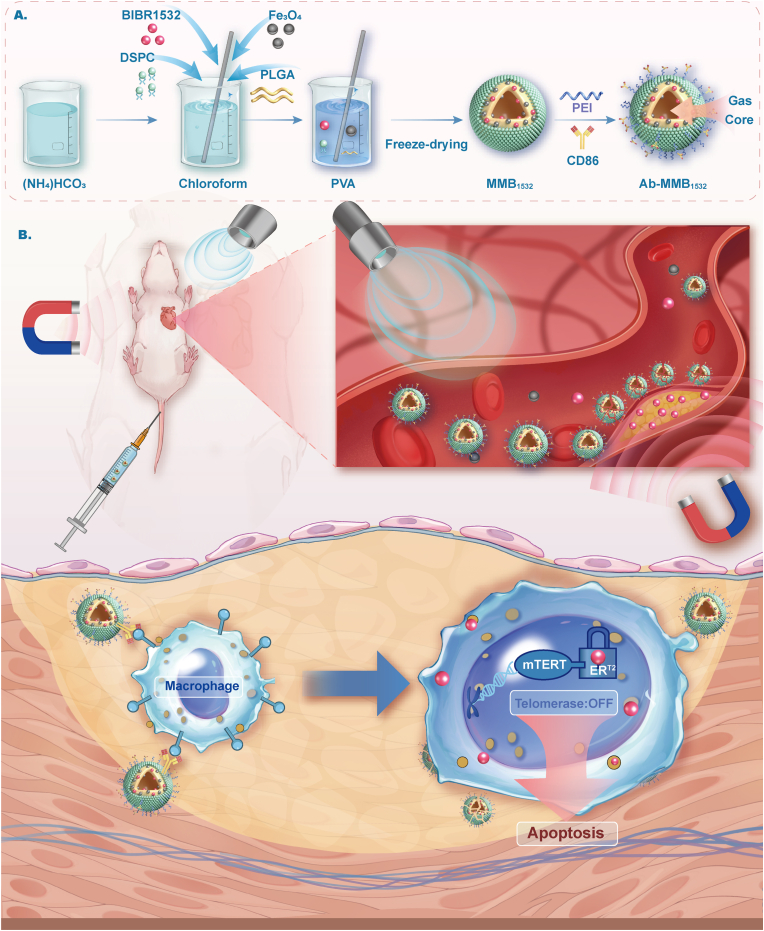
Schematic diagram displaying the Ab-MMB_1532_ fabrication, targeting, and its anti-AS properties.

Hence, based on the above background, we design a dual-target ultrasound microbubble delivery system (Ab-MMB_1532_) integrating CD86 antibody-mediated M1 targeting and magnetically guided navigation for site-specific delivery of the telomerase inhibitor BIBR1532 (Fig. 1). BIBR1532 is a synthetic, non-nucleoside, non-competitive, small-molecule telomerase inhibitor that can inhibit telomerase activity by specifically binding to the active site of TERT [28,29]. The results demonstrate that Ab-MMB_1532_ effectively targets and accumulates on M1 macrophages within AS plaques, driven synergistically by the dual guidance from an external magnetic field and CD86 antibody-mediated recognition. Subsequently, ultrasound-triggered cavitation at the lesion site facilitates the localized release of BIBR1532 into M1 macrophages. Integrated metabolomic and transcriptomic analyses reveal that BIBR1532 promotes macrophage apoptosis by inhibiting the TERT/NF-κB signaling axis. *In vivo* validation confirms the therapeutic superiority of Ab-MMB_1532_ in achieving optimal anti-AS efficacy.

## 2 Materials and Methods

### 2.1 Synthesis of MB_1532_, Ab-MB_1532_, MMB_1532_ and Ab-MMB_1532_

The MB_1532_ and MMB_1532_ were fabricated by a modified double emulsion solvent evaporation method [30]. Briefly, 50 mg of PLGA, 2.5 mg DSPC and a certain amount of BIBR1532 dissolved in 1.0 mL chloroform. Then 0.2 mL freshly prepared NH_4_HCO_3_ solution (60 mg/mL) was added to the above oil phase. The mixture was emulsified using an ultrasonic probe in an ice bath for 2 min, with a pulse mode of working 3 s and resting 3 s. Subsequently, the initial emulsified solution was slowly added into 5 mL PVA (4 %, w/v), and then homogenized for 5 min at 6000 rpm. Incorporate 10 mL of pre-cold deionized water into above obtained emulsion, and stir magnetically for 4 h at RT to evaporate the organic solvent. After that, Collect the precipitate by centrifugation at 6000 rpm and 4 °C for 10 min, followed by three washes with deionized water. Finally, resuspend the precipitate in 0.5 mL of deionized water, followed by vacuum freeze-drying for 24 h to obtain MB_1532_, which stored at 4 °C for use. Following the same method above, 3 mg oleic acid-modified Fe_3_O_4_ nanoparticles or 2 μL DiI was added into the oil phase to obtain the production of distinct entities: magnetically responsive MMB_1532_ and DiI-MB_1532_/MMB_1532_ with red fluorescent labeling.

The MB_1532_ and MMB_1532_ were further coated with polyethylenimine (PEI) to impart a positive charge, and then 20 μL of CD86 antibodies were added to obtain Ab-MB_1532_ and Ab-MMB_1532_ respectively, through electrostatic adsorption.

### 2.2 Characterization

The morphology of MB_1532_ and MMB_1532_ were analyzed by scanning electron microscope (SEM, Hitachi, Japan) and transmission electron microscope (TEM, JEOL, Japan). The particle size and zeta potential of MB_1532_ and MMB_1532_ were determined by Zetasizer NanoZS instrument (Malvern, UK). Vibrating sample magnetometer (VSM, Quantum Design, USA) was used to obtain the magnetization curve of MMB_1532_. To demonstrate whether Ab-MB_1532_ and Ab-MMB_1532_ successfully loaded CD86, fluorescence secondary antibodies (Dylight 649, Goat anti-mouse IgG) at the recommended concentration were incubated with MB_1532_ and MMB_1532_ for 10 min under protecting from the light at 4 °C, followed by observation under a confocal laser scanning microscope (Leica TCS SP8, Germany). In order to observe the magnetic response performance of MMB_1532_, a rectangular magnet (25× 10 × 5 mm, 1.2T) was positioned near a glass vial containing an MMB_1532_ aqueous solution, and a camera was employed to record the process of magnet attraction. To evaluate the stability of MB_1532_, its zeta potential and concentration were measured at 0 h, 4 h, 8 h, 12 h, 1 day, and 7 days after preparation.

Regarding the stability of CD86 antibodies electrostatically adsorbed onto Ab-MB_1532_ or Ab-MMB_1532_, both *in vivo* and *in vitro* experiments were conducted (detailed experimental procedures can be found in the supplementary materials).

### 2.3 Cell culture and M1 macrophage polarization stimulation

RAW264.7 murine macrophage cells were purchased from ATCC (cat #TIB-71), and cultured at 37 °C with 5 % CO_2_ in DMEM supplemented with 10 % FBS and 1 % penicillin-streptomycin. When reached 80 % confluence, adherent RAW264.7 cells were detached via scraping and then seeded in 6-well plates at a concentration of 3 × 10^5^ cells per well.

Primary murine macrophage bone marrow-derived macrophages (BMDM) were generated using a standardized and widely accepted protocol [31]. In summary, a single-cell suspension of bone marrow cells was obtained from the tibia, femur, and ilium bones. The cells were then cultured in RPMI medium 1640 supplemented with 10 % heat-inactivated FBS and 50 ng/mL recombinant mouse macrophage colony-stimulating factor (Gibco) for 7 days to induce differentiation.

For M1 macrophage polarization stimulation, 100 ng/mL of lipopolysaccharide (LPS) was added to the cell suspension mentioned above and mixed thoroughly, then seeded into a 6-well plate at a density of 3 × 10^5^ cells per well. The cells were incubated at 37 °C in a CO_2_ incubator for 24 h in preparation for the subsequent experiments.

For the purpose of omics analysis, M1 macrophages were grouped as follows: control cells, which were cultured without additional treatment, and experimental cells, which were exposed to 30 μM BIBR1532 for 24 h under the same conditions.

### 2.4 Untargeted metabolomics analysis

Samples were extracted with pre-cooled methanol: acetonitrile: H_2_O (2:2:1, v/v/v) and vortexed. Extracts were injected into the UPLC-MS/MS system for analysis. RAW data were converted to. mzXML format using ProteoWizard, followed by peak alignment, retention time correction, and peak area extraction using XCMS software. Data extracted by XCMS underwent metabolite structural identification and preprocessing, followed by quality assessment. Quality control (QC) samples were used for batch standardization. Differential analysis (*t*-test or ANOVA) was performed to determine p-values, adjusted p-values, fold change (FC), coefficient of variation (CV), and differential metabolites (p-value <0.05 and FC < 0.67 or FC > 1.5). Pathway analysis was conducted using MetaboAnalyst 5.0 metabolomics software.

### 2.5 RNA-seq and analysis

Total RNA was isolated from macrophages using TRIzol reagent (Thermo Fisher Scientific, USA) following the manufacturer's guidelines. The RNA quality and integrity were evaluated with an Agilent 2100 Bioanalyzer (Agilent Technologies, USA), and only samples with RNA integrity number (RIN) values above 7.0 were included for downstream processing. Sequencing libraries were prepared using the NEBNext Ultra RNA Library Prep Kit (New England Biolabs, USA) and sequenced on an Illumina NovaSeq 6000 platform to produce 150-bp paired-end reads. The resulting clean reads were mapped to the reference genome using HISAT2, and gene expression levels were calculated as fragments per kilobase of transcript per million mapped reads (FPKM). Differentially expressed genes (DEGs) analysis was conducted using DESeq2, with significant thresholds set at an adjusted p-value <0.05 and a fold change ≥2. Functional annotations and pathway enrichment were performed using Gene Ontology (GO) and Kyoto Encyclopedia of Genes and Genomes (KEGG) databases. Gene set enrichment analysis (GSEA) was conducted using the GSEA software. The results were visualized through the online platform (https://www.omicstudio.cn) and R software (version 4.4).

### 2.6 Optimal ultrasound therapy acoustic parameters *in vitro*

M1 macrophage cells in logarithmic growth phase were seeded into a 24-well plate at a density of 5 × 10^4^ cells per well, with 500 μl of culture medium in each well, and incubated at 37 °C in a CO_2_ incubator for 24 h. Different ultrasound parameters (intensity of 0.5, 0.8, 1.0, 1.2, 1.5, and 1.8 W/cm^2^; duty cycle of 10 %, 20 %, 30 %, 40 %, 50 %, and 60 %; treatment time of 10 s, 20 s, 30 s, 40 s, 50 s, and 60 s; Sonitron GTS Sonoporator, Japan), with untreated cells serving as a control. After another 24 h incubation, the cells were washed for three times using PBS, followed by staining by Live/Dead Cell Kit (KeyGEN, China). Briefly, the cells were incubated with Calcein-AM/PI solution (4 μM for each fluorescent probe in PBS) at RT for 45 min, followed by three washes with PBS, and imaging was performed under an in-verted fluorescence microscope (Laica DMi8, Germany). The excitation wavelengths for Calcein-AM and PI were 488 nm and 543 nm, respectively, with corresponding emission bands collected in different wavelength ranges, specifically 510–540 nm (green) and 570–620 nm (red).

### 2.7 CD86 antibodies and magnet-mediated BIBR1532 targeting transport *in vitro*

In order to assess the dual-targeted mediated biological effects, a modified inverted method was used, as we described previously [32]. In brief, this section of the experiment was divided into 6 groups (n = 4/group): the control group, free BIBR1532 group (30 μM), and 4 MB groups (MB_1532_, Ab-MB_1532_, MMB_1532_ and Ab-MMB_1532_). MB suspensions were prepared by mixing fresh complete DMEM medium with a MB concentration of 2.5 × 10^7^ particles per mL and a BIBR1532 content of 30 μM.

M1 macrophages were then seeded in a 24-well plate at a density of 5 × 10^4^ cells per well. When the cells reached approximately 70 % confluence, the medium was removed, and 3 mL of the prepared MB suspension was added to each well. Microseal 'B' Adhesive Seals (Bio-Rad, USA) was used to seal the cell culture plate, and magnets were placed at the bottom of plates in all groups, followed by mildly rotation and removement of magnets for 10 min. After that, the supernatant was removed, gently rinsed twice with PBS to remove free unbound MBs. Then, 500 μl of fresh culture medium was added to each well, followed by a 40 s (1.2 W/cm^2^ of intensity, 30 % of duty cycle) ultrasound treatment, and further overnight incubation. Protein expression was analyzed through Western blot (WB), and telomerase activity was assessed using a telomerase activity assay kit.

### 2.8 Parallel plate flow chamber assay

A home-made parallel plate flow chamber assay was used to analysis the magnetic & antibody targeting attachment, as we described previously [33]. In brief, 1 mL of the preprepared Dil-labeled MB solution (MB_1532_, Ab-MB_1532_, MMB_1532_ and Ab-MMB_1532_) at a concentration of 2.5 × 10^7^ particles per mL, was introduced into a vacuum parallel plate flow chamber (Glycotech 31-001, USA) by a stepping motor (Yuhui, China) via a capillary tube (*r* = 15 mm) at shear forces ranging from 6 to 48 dyn/cm^2^. Throughout the entire experimental procedure, a rectangular magnet was positioned above the chamber to capture the magnetic MBs (MMB_1532_ and Ab-MMB_1532_), while M1 macrophages were prepared below the chamber. Subsequently, the average red fluorescence intensity surrounding individual M1 macrophage was observed under the EVOS FL auto cell imaging system (Thermo Fisher Scientific, USA), allowing for the assessment of their targeting capability.

### 2.9 Targeting to AS plaques *in vivo*

ApoE^−/−^ mice (6-week-old) were purchased from Vital River Laboratory Animal Technology (Beijing, China). All animal experiments were approved by the Institutional Animal Care and Use Committee of Jinan University (No: 20210225-18). In order to induce the formation of AS, all ApoE^−/−^ mice were fed with a high-fat diet (HFD, including 41 % fat and 17 % protein) for 8 weeks prior to the experiment below. Saline, MMB_1532_, AB-MB_1532_ and Ab-MMB_1532_ were administered with a dose of 2 mg/kg via the tail vein, followed by a magnet placed on the trunk side of the heart level for 5 min in each group of mice. After 24 h, euthanasia was performed on the mice, and perfusion with pre-cooled PBS containing 4 % paraformaldehyde was conducted to remove unbound MBs. The separated aorta and major organs were imaged and quantified for fluorescence using the Xenogen IVIS 200 system.

### 2.10 Animals and treatment protocol

After 8 weeks of HFD feeding, randomly divided ApoE^−/−^ mice into 4 groups (n = 6/group): (1) saline group; (2) MMB_1532_ group (0.04 ng/kg BIBR1532); (3) Ab-MB_1532_ group (0.04 ng/kg BIBR1532); (4) Ab-MMB_1532_ group (0.04 ng/kg BIBR1532). All groups received weekly treatments for a consecutive duration of four weeks with 2 min ultrasound irradiation (1.5 W/cm^2^, 30 % duty cycle) at the anterior region of the mouse heart. Besides, for all groups, a magnet was initially placed on one side of the mouse torso for a 5 min period, after which the magnet was removed before initiating ultrasound irradiation. All were fed high-fat diet during the treatment.

After 4-week period of treatment, all mice were euthanized, and the aorta and heart were carefully dissected, with whole blood collected for hematological analysis. The aorta was longitudinally dissected for Oil Red O (ORO) staining to observe AS plaques macroscopically. After cardiac perfusion, multiple consecutive 8 μm-thick sections were prepared for hematoxylin and eosin (HE) staining, ORO staining, immunofluorescence (IF) staining and immunohistochemistry (IHC). Besides, HE staining was performed on major organs (heart, liver, spleen, lung, and kidney) to assess the bio-safety of the therapeutic agents.

### 2.11 Statistical analysis

Statistical analyses were performed using SPSS software version 17.0 (SPSS Inc., USA). Data from *in vitro* experiments are presented as mean ± standard error of the mean (SEM). Comparisons between two groups were conducted using a two-tailed Student's t-test. A p-value of less than 0.05 was considered statistically significant.

## 3 Results and Discussion

### 3.1 Synthesis and characterization of MB_1532_, Ab-MB_1532_, MMB_1532_, and Ab-MMB_1532_

**Fig. 2:**
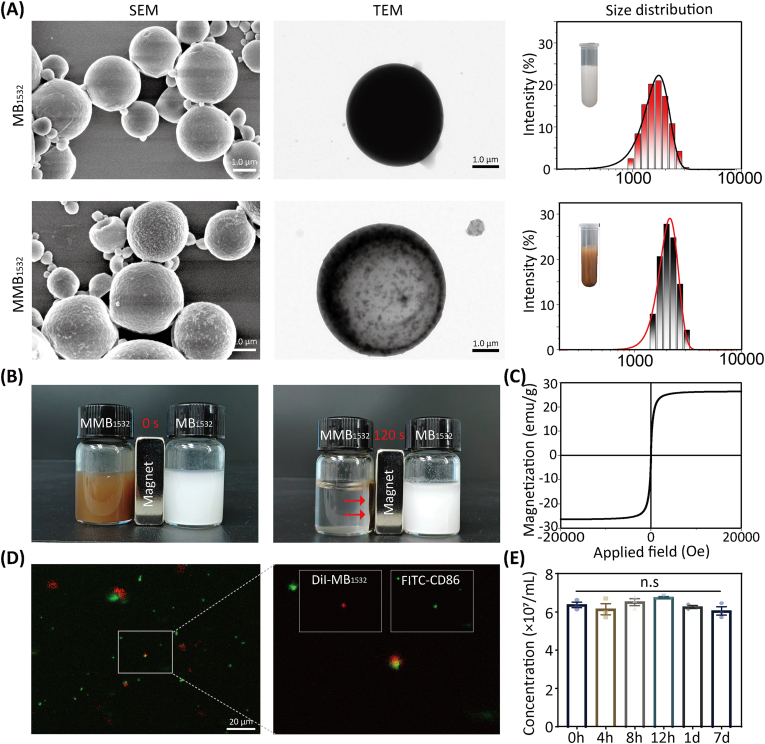
Fabrication and characterization of Ab-MMB_1532_. (**A**) SEM, TEM, and size distribution of MB_1532_ and MMB_1532_ respectively. (**B**) Comparative images of MMB_1532_ and MB_1532_ solutions before and after applying a magnetic field. (**C**) Field-dependent magnetization curve of MMB_1532_. (**D**) IF images of Ab-MB_1532_ show successful loading of CD86 antibodies (green) onto MB_1532_ (red). (**E**) Stability of the MB_1532_ over a 7 d period at 37 °C. (For interpretation of the references to colour in this figure legend, the reader is referred to the Web version of this article.)

Ab-MMB_1532_ was successfully prepared using a water/oil/water (W_1_/O/W_2_) double-emulsion method and electrostatic adsorption [30]. The SEM images showed that MB_1532_ and MMB_1532_ exhibited a hollow spherical morphology, which was particularly clear in the TEM images. Furthermore, in contrast to MB_1532_, numerous black Fe_3_O_4_ nanoparticles were encapsulated within the bubble shells of Ab-MMB_1532_ (Fig. 2A). The average particle sizes of MB_1532_ and MMB_1532_ measured by dynamic light scattering were 1398 ± 17.72 nm and 2291 ± 157.09 nm, respectively, with their polydispersity indices at 0.26 ± 0.07 and 0.23 ± 0.10, respectively (Fig. 2A–Table S1). Before PEI modification, the zeta potential of MB_1532_ and MMB_1532_ were −18.33 ± 0.86 mV and −21.23 ± 1.46 mV, respectively. However, following positive-charge modification, the potentials of MB_1532_ and MMB_1532_ significantly increased to 14.43 ± 1.34 mV and 20.27 ± 1.12 mV, respectively; after incubation with CD86 antibodies, their potentials slightly decreased to 7.62 ± 0.36 mV and 9.77 ± 0.46 mV, respectively (Fig. S1). In addition, during 7 days follow-up in phosphate buffered saline (PBS) solution, the concentration and zeta potential of MB_1532_ remained almost unchanged (*p* > 0.05), suggesting that MB_1532_ had remarkable stability and dispersion characteristics (Fig. 2E–S2).

In the presence of a magnetic field, the uniform dispersion of MMB_1532_ in PBS rapidly migrated towards the orientation of the magnetic field within 120 s (Fig. 2B). Notably, the field-dependent magnetization curve indicated that MMB_1532_ possessed exceptional superparamagnetic properties, with a magnetization saturation of 26.3 emu/g (Fig. 2C). Subsequently, immunofluorescent (IF) staining was performed to examine whether Ab-MMB_1532_ was successfully loaded with CD86 antibodies. As shown in Fig. 2D, Ab-MMB_1532_ simultaneously emitted both red fluorescence (DiI-labeled MB_1532_) and green fluorescence (FITC-conjugated CD86) from the bubble shells, demonstrating that CD86 antibodies were bound to the surface of Ab-MMB_1532_ through electrostatic adsorption. Furthermore, our *in vitro* and *in vivo* experiments demonstrated that CD86 antibodies electrostatically adsorbed onto MMB_1532_ or MB_1532_ remained relatively stable under physiological conditions, with a dissociation rate of less than 25 % within 2 h (22 % for *in vivo*, 13 % for *in vitro*; Fig. S3). The drug-loading rates of MB_1532_ and MMB_1532_ were characterized using UV–Vis absorption spectroscopy (Fig. S4) [34].

### 3.2 BIBR1532 inhibits M1 macrophage proliferation by reducing telomerase activity

Macrophages, particularly M1 macrophages, are one of the major inflammatory cells in atherosclerotic lesions [12]. The formation of foam cells by macrophages leads to AS [35]. We demonstrated a high expression of CD86 (a marker of M1 macrophages) within atherosclerotic plaques in Apoe^−/−^ mice using IF staining (Fig. S5). Therefore, we selected RAW264.7 cells and BMDMs, and successfully polarized them into M1 macrophages for subsequent experiments (Figs. S6 and S7).

**Fig. 3:**
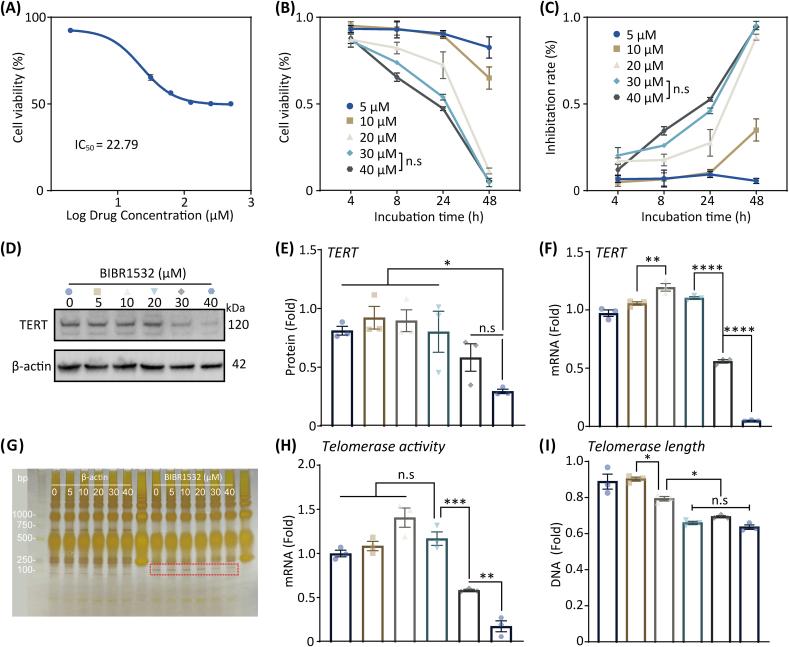
Effect of BIBR1532 on M1 macrophages (RAW264.7 derived). (**A**) The IC_50_ value of BIBR1532 was evaluated using CCK8 assays. (**B, C**) Cell viability and inhibition rate were evaluated by CCK8 assay at different time points. (**D, E**) The expression and quantitative analysis of TERT protein by WB. (**F**) The q-PCR analysis of TERT gene expression. (**G, H**) Telomerase activity was detected using a fluorescent RT-qPCR assay kit, and visualizing the results using a protein silver staining kit. (**I**) Telomerase length was detected using a telomere length detection kit.

BIBR1532 has been widely used in studies on telomerase function, with reports suggesting that it effectively inhibits cell viability in various cancer-cell types [28]. However, its use in studies on macrophages remains relatively limited. Therefore, we investigated the half-inhibitory concentration (IC_50_) of BIBR1532 in M1 macrophages. Based on the 48 h CCK8 dose-response curve, the IC_50_ values of BIBR1532 for RAW264.7 cells and BMDMs were calculated as 22.79 μM and 23.33 μM, respectively (Fig. 3A, S8A). To further investigate the potential cell-suppressive effects of BIBR1532 in M1 macrophages, different concentrations of BIBR1532 (5, 10, 20, 30, and 40 μM) were added to both RAW264.7 cells and BMDMs. The results demonstrated that BIBR1532 significantly inhibited cell proliferation in a dose-dependent manner (Fig. 3B–C, S8B). However, no significant difference in cell-inhibition rates was observed between the drug concentrations of 30 μM and 40 μM for RAW264.7 cells (Fig. 3C), and between 20 μM, 30 μM and 40 μM for BMDMs (Fig. S8B).

BIBR1532 inhibited cell proliferation by reducing TERT expression. WB analysis revealed that the lowest TERT expression occurred at 40 μM BIBR1532 in RAW264.7 cells (Fig. 3D and E) and 20 μM in BMDMs (Fig. S8C). However, no significant differences were observed between 30 μM and 40 μM (RAW264.7 cells) or within the 20–40 μM range (BMDMs). Similarly, the mRNA expression of TERT (Fig. 3F–S8D) provided consistent results, indicating a significant decrease at a BIBR1532 concentration at 30 μM (RAW264.7 cells) and 20 μM (BMDMs). We further investigated telomerase activity, and the results from the telomerase-activity assay kit and silver staining (Fig. 3G and H) showed that at a BIBR1532 concentration of 30 μM, M1 macrophage telomerase activity was significantly reduced, whereas at a concentration of 10 μM, telomerase activity was slightly elevated. We speculated that this may be owing to stress-responsive genes, which were transcribed at low levels under normal conditions and were robustly induced in response to stress [36]. In addition, we investigated changes in telomere length in M1 macrophages (Fig. 3I). At lower concentrations (10 μM), telomere length began to decrease, which may be owing to the partial inhibition of telomerase, whereas at 20 μM, the most pronounced reduction in both telomerase activity and telomere length was observed. However, at higher concentrations (30 and 40 μM), telomere length stabilized, which may have been the result of a plateau effect in telomerase inhibition, where further increases in BIBR1532 did not lead to a significant additional decrease in telomere length. This suggests that telomerase activity and telomere length were regulated by different inhibition thresholds, with telomere length being more sensitive to lower concentrations of BIBR1532. Therefore, these results consistently indicated that BIBR1532 inhibits cell proliferation by reducing telomerase activity, particularly at concentrations of 30 μM (RAW264.7 cells) and 20 μM (BMDMs). However, the underlying mechanisms require further investigation.

**Fig. 4:**
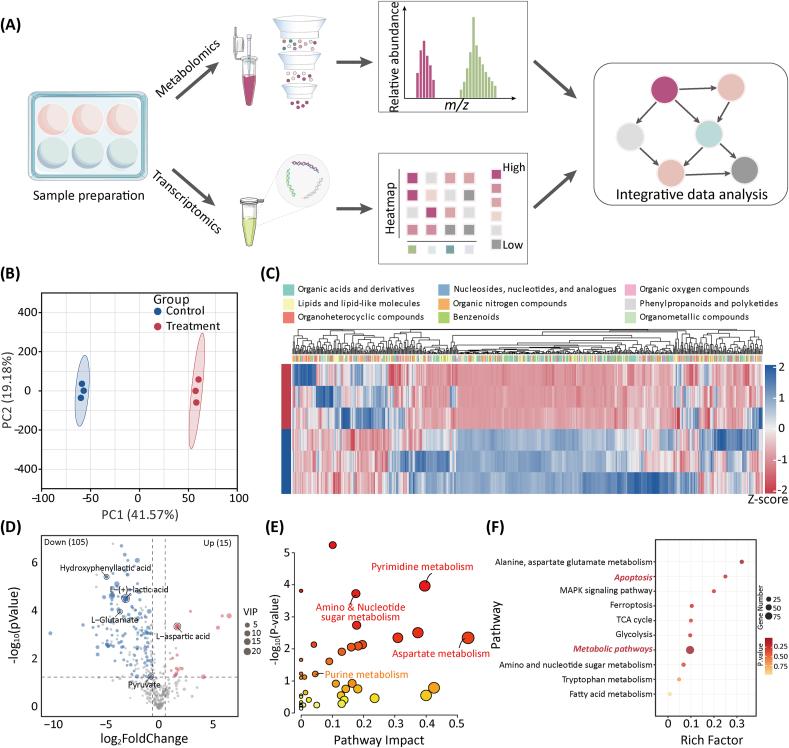
The metabolomic analysis of BIBR1532 on M1 Macrophages. (**A**) Schematic of metabolomic and transcriptomic sample extraction and analysis. (**B**) PCA score plot showing differences between the control and the treatment groups. (**C**) Heatmap for the distribution of metabolites. (**D**) Volcano plot demonstrating altered metabolite levels. Fold Change <0.5/> 2 and P-value <0.05 (**E**) Pathway analysis of significant differential metabolites. (**F**) KEGG pathway enrichment analysis based on the LC-MS metabolomics assay.

Multi-omics analysis has become a powerful tool in biomedical research, enabling the breakdown of complex biological systems by integrating various molecular data, thereby providing a comprehensive view for uncovering mechanisms [37]. Therefore, we performed metabolomic and transcriptomic analyses of M1 macrophages before and after treatment with BIBR1532 (Fig. 4A).

### 3.3 Metabolomics analysis

Metabolomics has led to the discovery of new biomarkers and a better mechanistic understanding of diseases with applications in precision medicine [38]. We used ultra-performance liquid chromatography-tandem mass spectrometry to assess the metabolomic characteristics of M1 macrophages treated with and without BIBR1532. As shown in the principal component analysis (PCA) score plot (Fig. 4B), the two groups exhibited clear separation trends. We identified 598 metabolites between the two groups (Fig. 4C), the majority of which were organic acids and derivatives (33.04 %) and lipids and lipid-like molecules (21.43 %). After filtering (fold change <0.5/> 2 and P-value <0.05), 120 differential metabolites (15 upregulated and 105 downregulated) were identified in the treatment group compared to the control group, as shown in the volcano plot (Fig. 4D). Among these, L-(+)-lactic acid (associated with macrophage inflammation), hydroxyphenyllactic acid (related to oxidative stress), and L-glutamate and pyruvate (related to energy metabolism) were downregulated after BIBR1532 treatment. These differential metabolites were further analyzed for pathway enrichment using MetaboAnalyst. The results indicated that the most affected pathways primarily involved pyrimidine & purine, aspartate, and amino & nucleotide sugar metabolism (Fig. 4E). Notably, compared to the control group, L-glutamine (approximately 0.17-fold) and uridine monophosphate (UMP, approximately 0.13-fold), which are involved in purine and pyrimidine metabolism, were significantly downregulated after BIBR1532 treatment, whereas L-aspartic acid (approximately 3.29-fold), which is involved in aspartic acid metabolism, was significantly upregulated. Nucleotide levels control telomere lengthening in human cells [39] and influence cellular metabolism. In other words, after BIBR1532 treatment, telomerase activity in M1 macrophages was inhibited, leading to a halted purine, pyrimidine, and nucleoside metabolism; impaired cellular-energy metabolism; reduced oxidative stress; and the resolution of inflammation. Notably, aspartic-acid metabolism was enhanced. Studies have indicated that the homeostasis of aspartic acid and asparagine is crucial for cellular fate [40]. KEGG pathway-enrichment analyses (Fig. 4F) showed that the altered metabolites were related to metabolic pathways and apoptosis. Therefore, these findings suggest that BIBR1532 inhibits telomerase activity in M1 macrophages, affecting cellular metabolic processes (including energy metabolism, oxidative stress, and inflammation), thereby promoting apoptosis.

### 3.4 Transcriptomics analysis

**Fig. 5:**
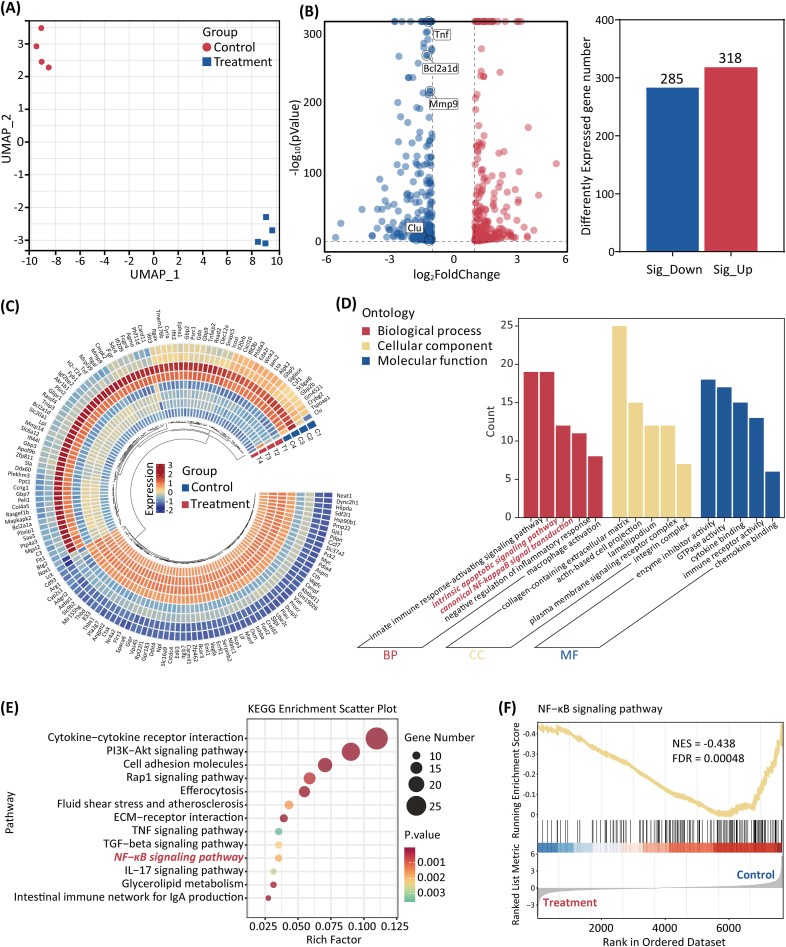
The transcriptomic analysis of BIBR1532 on M1 Macrophages. (**A**) PCA plot of RNA-seq data. (**B**) Volcano plot showing significantly upregulated (red) and downregulated (blue) genes, with a bar chart of their counts. (**C**) Heatmap of DEGs. (**D**) The top 5 terms in each GO analysis. (**E**) KEGG pathway analysis for differentially expressed genes (DEGs). (**F**) GSEA of the downregulated signal pathway “NF-κB”. NES, normalized enrichment score; FDR, false discovery rate. (For interpretation of the references to colour in this figure legend, the reader is referred to the Web version of this article.)

Transcriptomics is a vital tool for investigating gene-expression profiles and understanding gene functions [41]. RNA-seq transcriptomic analysis was conducted to examine the differences in gene expression induced by the treatment to explore the molecular mechanisms by which BIBR1532 modulates the behavior of M1 macrophages. PCA demonstrated clear clustering of the three biological replicates within each group, highlighting the distinct gene-expression profiles between the Control and Treatment groups (Fig. 5A). In the Treatment group, 318 DEGs were upregulated and 285 were downregulated compared with those in the Control group (Fig. 5B). Notably, genes associated with macrophage inflammation, including *Tnf* and *Mmp-9*, and anti-apoptotic genes, such as *Clu* and *Bcl2a1d*, were significantly downregulated following BIBR1532 treatment. Some DEGs were visualized using a heatmap (Fig. 5C). GO analysis (Fig. 5D) was performed on the biological processes (BPs), cellular components (CCs), and molecular functions (MFs) of the DEGs [42]. The results showed that the BP category was primarily enriched in the intrinsic apoptotic signaling pathway and canonical NF-κB signal transduction. The CCs were significantly enriched in cell adhesion-related pathways, including the collagen-containing extracellular matrix and actin-based cell projections. The MF category was mainly enriched in enzyme inhibition and cytokine pathways. Additionally, KEGG pathway analysis (Fig. 5E) revealed that the DEGs were enriched in pathways related to cytokine–cytokine receptor interaction and PI3K-Akt signaling pathway, with notable enrichment also observed in the NF-κB signaling pathway. Finally, GSEA results (Fig. 5F) indicated that the NF-κB signaling pathway was preferentially suppressed in the treatment group.

### 3.5 Omics integration analysis

**Fig. 6:**
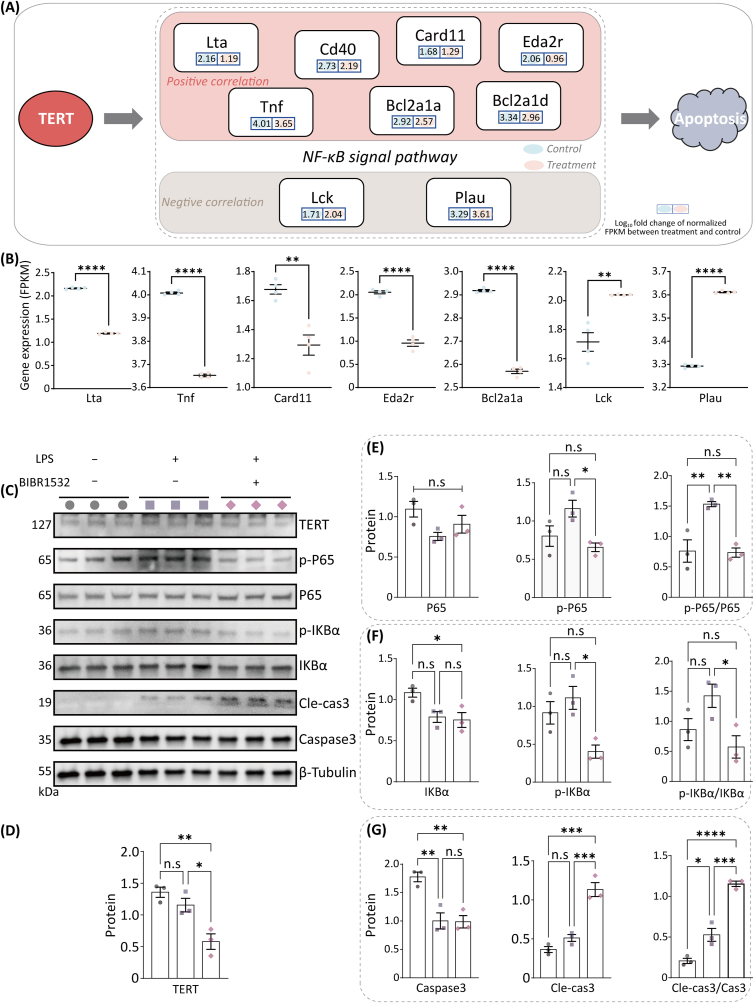
The effect of BIBR1532 on TERT/NF-κB signaling axis. (A) Schematic diagram of the correlation between TERT, NF-κB signaling pathway and cell apoptosis. (**B**) Expression of genes in the NF-κB signaling pathway. (**C**) The expression and quantitative analysis of TERT, p-P65, P65, p-IκBα, IκBα, caspase-3 and cleaved caspase-3 protein by WB. (**D**) The quantification histogram of TERT protein expression normalized by β-Tubulin; and (**E-G**) the quantification histogram of p-P65, P65, p-IκBα, IκBα, caspase-3 and cleaved caspase-3 protein expression normalized by β-Tubulin, along with their respective ratios. Data are presented as mean ± SEM. ∗∗*p* < 0.05, ∗∗∗*p* < 0.01, ∗∗∗∗*p* < 0.001.

Based on the metabolomic and transcriptomic analyses above, we conclude that BIBR1532 inhibited telomerase activity and promoted apoptosis in M1 macrophages through the downregulation of the NF-κB signaling pathway. Transcriptomic analyses (Fig. 6A, Table S3) revealed nominal positive correlations between TERT expression and seven NF-κB pathway components (Pearson's *r* = 0.42–0.67), while two genes exhibited inverse correlations (*r* = −0.38 to −0.55). However, statistical significance was not achieved (*p*-value >0.05), possibly because of the limited sample size; thus, further validation in larger cohorts is warranted. The FPKM expression levels of these pathway-related genes are shown in Fig. 6B.

Subsequently, WB analysis was performed to validate the functional modulation of this pathway (Fig. 6C). The results demonstrated that BIBR1532 treatment effectively suppressed TERT-protein expression (approximately 50 % reduction), whereas LPS stimulation failed to induce TERT-protein upregulation (Fig. 6D). To investigate whether BIBR1532 regulates inflammation and enhances apoptosis through the NF-κB signaling pathway, we systematically assessed the phosphorylation status of IκBα/P65 and the cleavage of caspase-3. The results showed that LPS stimulation significantly activated the typical NF-κB signaling pathway, with an increase in the ratio of p-IκBα/IκBα and p-P65/P65 (*p* < 0.01 vs. control). Notably, BIBR1532 treatment inhibited the phosphorylation of IκBα (reduced by 36 %) and P65 (reduced by 56 %) in LPS-induced M1-polarized macrophages (Fig. 6E and F), while enhancing the generation of cleaved caspase-3 (increased by 2.2-fold, Fig. 6G).

Additionally, flow cytometry was used to further investigate the effects of BIBR1532 on apoptosis in M1 macrophages. The results showed that the percentage of annexin-V-positive cells were significantly increased from 5.99 % to 9.75 % (*p* < 0.05) after treating M1 macrophages with 10 μM of BIBR1532 for 48 h (5.42 % of cells in early apoptosis and 4.33 % in late apoptosis). Furthermore, as the concentration of BIBR1532 increased, the percentage of annexin-V-positive cells further increased (Fig. S9A). We also investigated the cellular-senescence status using a SA-β-Gal staining kit. The number of SA-β-Gal-positive cells gradually increased as the concentration of BIBR1532 increased, especially at 30 μM. However, when the concentration of BIBR1532 reached 40 μM, we did not observe the expression of SA-β-Gal. We speculated that this was due to the toxic effects of BIBR1532, leading to cell death (Fig. S9B).

Our findings established that BIBR1532 exerted pro-apoptotic effects in M1 macrophages through a tripartite regulatory effect: (i) inhibition of telomerase activity (TERT downregulation confirmed at both the mRNA and protein levels); (ii) suppression of NF-κB signaling, disruption of nucleotide metabolism, and amino-acid homeostasis; and (iii) activation of caspase-dependent apoptosis.

### 3.6 Dual-target and pro-apoptotic efficacy of Ab-MMB_1532_*in vitro*

Before assessing the biological effects of Ab-MMB_1532_, we evaluated whether the MBs without drug loading had any impact on M1 macrophage activity. The CCK-8-assay data revealed that M1 activity consistently remained above 90 % following co-incubation with various concentrations of MBs at different time points. This suggests that MBs do not exhibit significant cytotoxicity towards M1 macrophages, demonstrating excellent biocompatibility (Fig. S10).

**Fig. 7:**
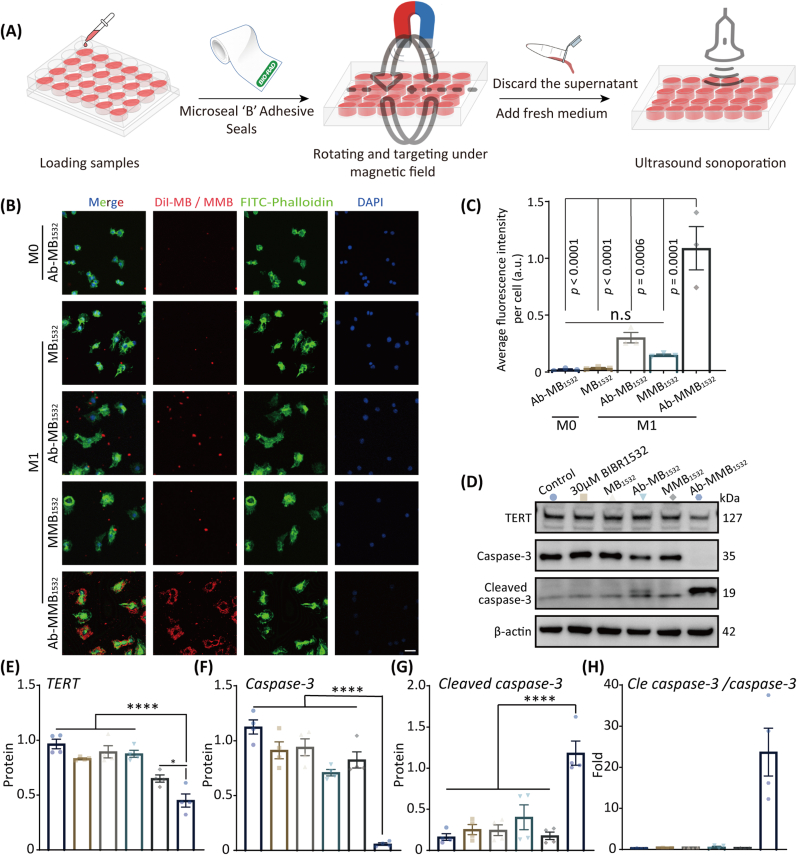
The dual-targeting capabilities and pro-apoptotic potential of Ab-MMB_1532_. (**A**) Schematic of cell therapy *in vitro*. (**B**) Assessment Ab-MMB_1532_ targeting specificity by IF. Red represents MB/MMB stained with DiI, green represents FITC-labeled phalloidin staining the macrophage cytoskeleton, and blue represents DAPI staining of cell nuclei. Scale bar: 25 μM (**C**) Semiquantitative analysis of average fluorescence intensity. (**D**) The expression and (**E-H**) quantitative analysis of TERT, caspase-3 and cleaved caspase-3 protein by WB. ∗p < 0.05, ∗∗p < 0.01, and ∗∗∗p < 0.001. (For interpretation of the references to colour in this figure legend, the reader is referred to the Web version of this article.)

Many studies have demonstrated that delivery systems based on dual ligands are effective compared to single ligands or non-functionalized nanomedicines [43]. This study employed active targeting with CD86 antibodies and magnetic targeting via an external magnetic field to investigate the biological effects of Ab-MMB_1532_ on M1 macrophages *in vitro*. As shown in Fig. 7A, we employed an improved inverted method to simulate *in vitro* dual-target-mediated biological effects, as described previously [32].

The findings indicated a substantial concentration of Ab-MMB_1532_ with dual targeting around M1 macrophages, resulting in a targeting-efficiency enhancement of more than 50 % compared with single-targeting or non-targeting agents (Fig. 7B and C). Additionally, to investigate the specificity targeting of Ab-MB_1532_, we used M0 macrophages as negative controls. Given the nearly absent expression of CD86 in M0, almost no Ab-MB_1532_ accumulation was observed around these cells (Fig. 7B). This indicated the specific targeting capability of the Ab-MB_1532_ that we prepared. Therefore, dual-target delivery systems can significantly improve the specific targeting efficiency to M1 macrophages.

After targeting M1 macrophages, we subsequently applied the determined ultrasound parameters (Fig. S11) for *in vitro* therapy. The WB results demonstrated that dual-targeting Ab-MMB_1532_ effectively suppressed TERT protein expression in M1 macrophages (Fig. 7D and E). TERT is a crucial catalytic subunit of telomerase and its high expression can increase proliferation, anti-apoptosis, and invasion [44]. Therefore, the suppression of TERT expression can inhibit the proliferation of M1 macrophages and promote apoptosis. Furthermore, based on the aforementioned omics-analysis results, we examined the expression of apoptosis-related proteins caspase-3 and cleaved caspase-3. As anticipated, the results demonstrated that Ab-MMB_1532_ significantly promoted the expression of cleaved caspase-3 while reducing the expression of caspase-3 (Fig. 7F–H).

In summary, our data showed that Ab-MMB_1532_ could efficiently and specifically target M1 macrophages. Under ultrasound cavitation, the released BIBR_1532_ could inhibit macrophage proliferation and promote apoptosis by suppressing TERT-protein expression, which contributes to preventing the progression of plaque lesions caused by M1 macrophage activity.

### 3.7 Parallel plate flow-chamber assay and targeting capability of MBs *in vivo*

**Fig. 8:**
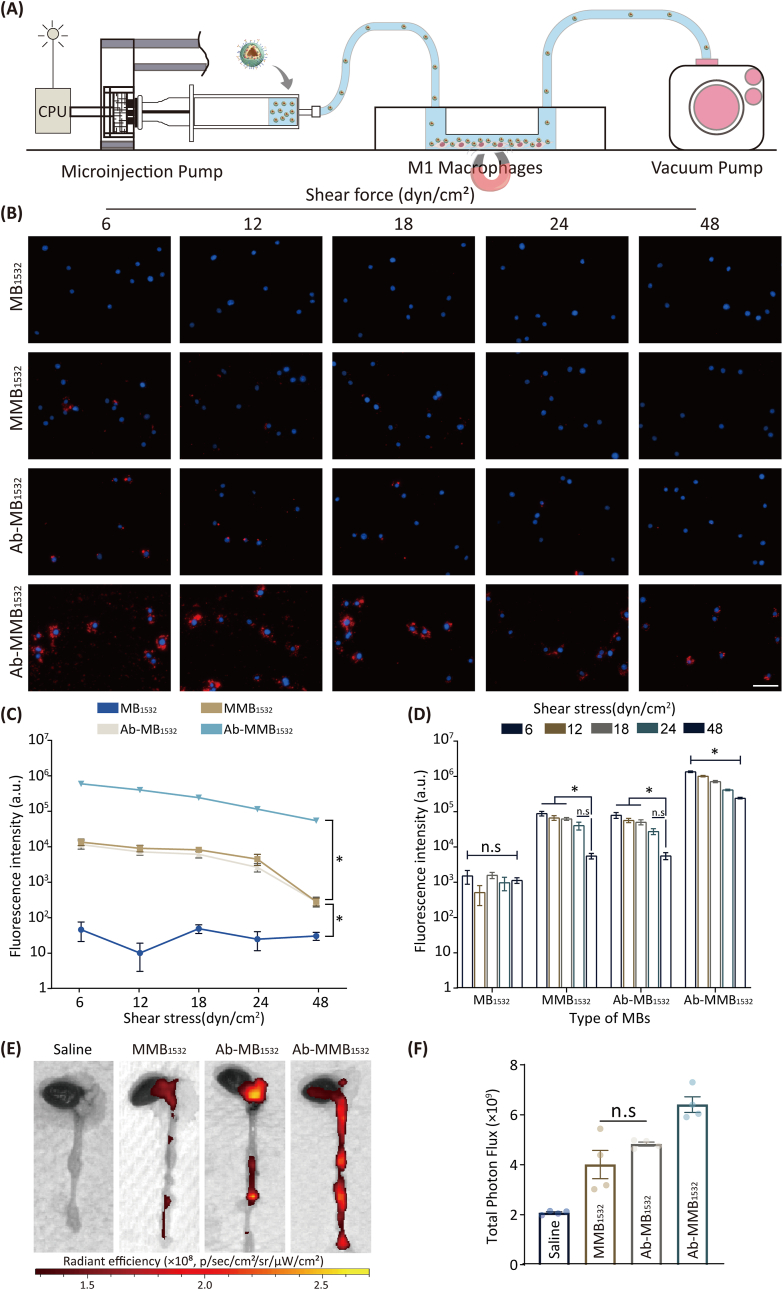
Parallel plate flow chamber assay and targeting AS plaques in ApoE^−/−^ mice. (**A**) Schematic of the custom-designed parallel plate flow chamber assay. (**B**) Fluorescence imaging of MB capture by M1 macrophages under varying shear forces in the presence of an applied magnetic field (B ≈ 1.2 T). Red represents MBs modified by DiI, and blue represents DAPI. Scale bar: 50 μm. (**C, D**) Semiquantitative analysis of average fluorescence intensity. (**E**) Representative ex vivo fluorescence images and (**F**) quantitative data of DiD fluorescent signals accumulated in the aorta 24 h post-injection (*n* = 4, mean ± SD, ∗*p* < 0.05, n.s: no significance). (For interpretation of the references to colour in this figure legend, the reader is referred to the Web version of this article.)

The targeting efficiency of different MB groups was assessed using a custom-designed parallel-plate flow chamber under varying shear forces (6 dyn/cm^2^ to 48 dyn/cm^2^; Fig. 8A) to mimic the magnetic responsiveness and antibody-targeting capabilities of MBs *in vivo*. As illustrated in Fig. 8B–D, for the four types of MBs investigated (MB_1532_, MMB_1532_, Ab-MB_1532_, and Ab-MMB_1532_), an increase in shear force resulted in a decrease in the number of captured MBs under the influence of the magnetic field. Notably, the non-targeted group (MB_1532_) exhibited minimal capture of MBs, with no statistically significant differences observed across the various shear forces (Fig. 8D). Conversely, the Ab-MMB_1532_ group, which possessed dual-targeting capability, demonstrated the highest MB-capture rate. Even under the highest shear force, it outperformed both the single-target (MMB_1532_ and Ab-MB_1532_) and non-target (MB_1532_) groups (Fig. 8C). Our data suggest that even in high-flow regions such as the aortic arch, a common site for AS, dual-target Ab-MMB_1532_ demonstrated unique potential for targeted therapeutic applications.

Subsequently, we investigated the targeting capability of the *in vivo* administration of MBs to AS plaques in Apoe^−/−^ mice. After a 24-h post-injection period, bright red fluorescence was prominently observed in the aortic arch and abdominal aorta, which are regions prone to plaque development (Fig. 8E). Simultaneously, the Ab-MMB_1532_-administration group exhibited a significantly higher average fluorescence intensity in the aorta than the other three groups (*p* < 0.05), with no significant differences observed among the single-target groups (MMB_1532_ *v.s* Ab-MB_1532_, *p* > 0.05, Fig. 8F). Additionally, the fluorescence primarily accumulated in the heart, liver, spleen, and lungs (Fig. S12).

### 3.8 Acute toxicity of Ab-MMB_1532_*in vivo*

Before evaluating the anti-AS effects of Ab-MMB_1532_ *in vivo*, we evaluated its acute toxicity. After a 4-h intravenous tail-vein injection of Ab-MMB_1532_, the blood and major organs of the mice were collected, with a PBS injection serving as a control. Hematological analysis revealed no significant differences in red blood cells (RBCs), white blood cells, platelets (PLT), lymphocytes (Lym), neutrophils, or hemoglobin levels between the Ab-MMB_1532_ and PBS groups, and all parameters remained within normal physiological ranges (Fig. S13A). Hematoxylin and eosin (HE)-staining results demonstrated no apparent pathological changes in major organs such as the heart, liver, spleen, lung, and kidney (Fig. S13B). These findings suggest that Ab-MMB_1532_ exhibits good biocompatibility and is a promising candidate for AS treatment.

### 3.9 Therapeutic efficacy against AS

**Fig. 9:**
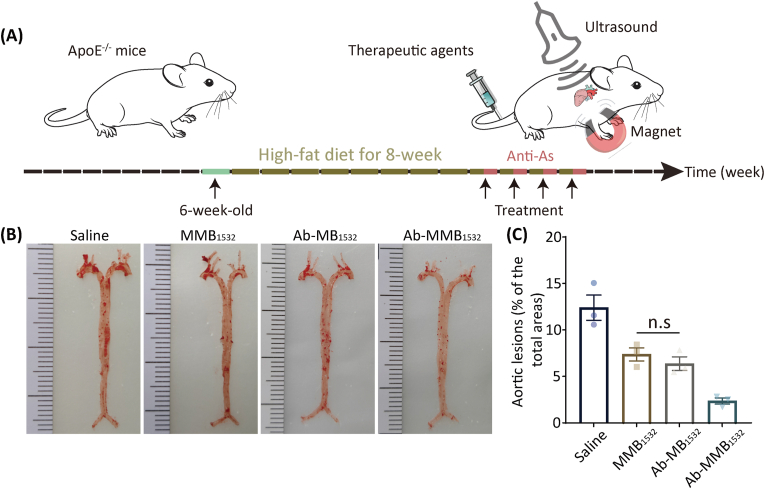
Therapeutic efficacy of AS in ApoE^−/−^ mice. (**A**) Schematic representation of the treatment protocol in this study. (**B**) Representative Oil-red O staining of the entire aorta. (**C**) Quantitative analysis of lesion area. Data are expressed as mean ± SEM (*n* = 3 per group). n.s: no significance. (For interpretation of the references to colour in this figure legend, the reader is referred to the Web version of this article.)

Based on these promising results, the therapeutic effects of Ab-MMB_1532_ were evaluated in an AS mouse model. Following the treatment protocol shown in Fig. 9A, six-week-old mice were subjected to an eight-week high-fat diet to induce pathological AS. At the end of the treatment period, the entire aorta was stained with Oil Red O (ORO). As shown in Fig. 9B, the saline group displayed the largest lesion area as a percentage of the total aortic area, reaching 12.41 %. MMB_1532_ and Ab-MB_1532_ treatments resulted in a moderate reduction in plaque areas by 7.37 % and 6.38 %, respectively. Notably, the Ab-MMB_1532_-treatment group exhibited a substantial decrease in the lesion area as a proportion of the total aortic area, reducing it to 2.35 % (Fig. 9C). These findings suggest that Ab-MMB_1532_ has a strong anti-AS effect owing to its dual-target properties.

**Fig. 10:**
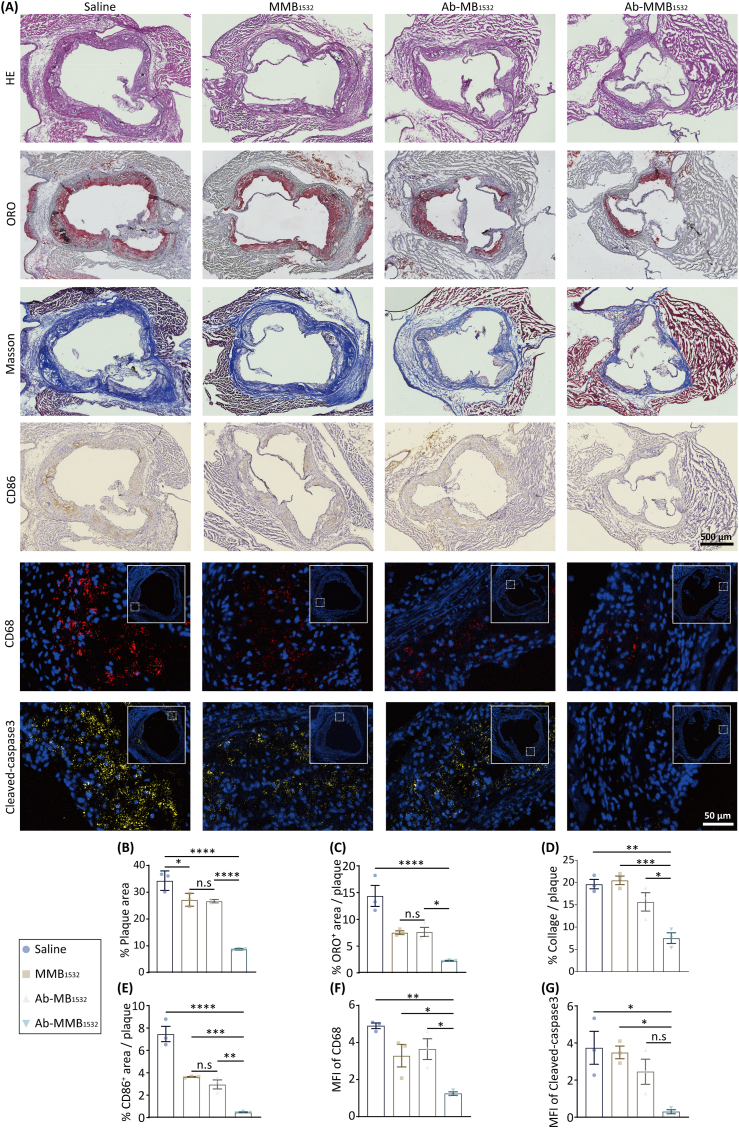
The anti-AS effect of Ab-MMB1532. (A) Representative sections of aortic root from each group of mice stained with HE, ORO, Masson's trichrome, anti‐CD86 antibody, anti‐CD68 antibody and anti‐cleaved caspase-3 antibody. (**B**) HE is quantified as a percent of lumen area. (**C**) ORO, (**D**) Masson and (**E**) CD86 are quantified as a percent of total plaque area. (**F, G**) Quantitative analysis of mean fluorescence intensity (MFI) of CD68 and cleaved caspase-3. Data are expressed as mean ± SEM (*n* = 3 per group). ∗*p* < 0.05, ∗∗*p* < 0.01, ∗∗∗*p* < 0.001 and n.s, no significance.

Subsequently, frozen aortic-root sections were systematically analyzed by HE staining, ORO staining, Masson's trichrome staining, IHC, and IF. As shown in Fig. 10A and B, HE staining of the aortic root in the saline group revealed numerous large plaques and necrotic cores. In contrast, both the MMB_1532_- and Ab-MB_1532_-treated groups showed significant attenuation of AS-plaque formation, with plaque-area reductions of approximately 20.7 % and 22.3 % (*p* < 0.05), respectively, compared to the controls. Notably, the Ab-MMB_1532_-therapy group exhibited the most pronounced therapeutic efficacy, achieving a remarkable reduction of 74.6 % in AS plaque (*p* < 0.001).

ORO staining revealed significantly reduced lipid accumulation within AS plaques in the therapeutic-agent-treated groups (MMB_1532_, Ab-MB_1532_, and Ab-MMB_1532_) compared to saline controls. Quantitative analysis demonstrated that plaque-lipid deposition accounted for 14.4 % of the total plaque area in the saline-treated group. In contrast, the MMB_1532_- and Ab-MB_1532_-treated groups exhibited 46.8 % and 61.6 % (*p* < 0.05) reductions in lipid deposition relative to controls, respectively. Interestingly, Ab-MMB_1532_ treatment achieved the most potent lipid-lowering efficacy, with 81.2 % attenuation (*p* < 0.001) (Fig. 10A–C). Furthermore, the analysis of serum LDL, CHO, and TG levels in each group revealed that the anti-AS effect of the therapeutic agents was not achieved through serum lipid reduction (Figs. S14D–F).

Masson's trichrome staining (Fig. 10A–D) revealed significant differences in collagen deposition between the groups. The saline (19.6 %), MMB_1532_ (20.5 %), and Ab-MB_1532_ (15.7 %) groups showed progressively higher collagen content, whereas the Ab-MMB_1532_ group exhibited the lowest collagen content (7.5 %). Furthermore, *in vivo* cleaved caspase-3 IF quantification (Fig. 10A–G) revealed the lowest apoptotic activity in the Ab-MMB_1532_ group, despite demonstrating the strongest pro-apoptotic effects *in vitro* (Fig. 7F–H). These findings may reflect the dynamic process of tissue remodeling and resolution of inflammation following Ab-MMB_1532_ treatment.

Previous studies have shown that M1 macrophages aggravate atherosclerosis progression by stimulating inflammation and lipid engulfment [45]. In this study, we investigated whether Ab-MMB_1532_ modulates macrophage infiltration within AS plaques to achieve therapeutic effects without affecting pre-existing lipid levels. IF analysis of the macrophage marker CD68 (Fig. 10A–F) revealed a significant reduction in macrophage infiltration into the plaques following treatment, particularly in the Ab-MMB_1532_ group. Consistently, the infiltration of M1 macrophages, as assessed by IHC (Fig. 10A–E), was markedly reduced to approximately 6.3 % of that of the saline group (*p* < 0.001). These results suggest that Ab-MMB_1532_ effectively inhibits macrophage infiltration, particularly that of pro-inflammatory M1 macrophages, thereby exerting an anti-AS effect.

In summary, our experimental findings demonstrate that Ab-MMB_1532_ effectively attenuates AS by reducing lipid deposition, inhibiting macrophage infiltration, particularly of M1 macrophages, and improving the inflammatory microenvironment within plaques, thereby reducing the plaque area.

### 3.10 Biosafety assessment

After a four-week treatment period, we assessed the potential side effects associated with various treatment modalities. Our findings revealed no statistically significant differences in the hematological parameters (RBC, Lym, and PLT) or serum lipid levels (LDL, CHO, and TG) between the MMB_1532_, Ab-MB_1532_, Ab-MMB_1532_, and saline groups (*p* > 0.05, Figs. S14A–F). The HE staining results also exhibited no notable pathological changes in the major organs (heart, liver, spleen, lung, and kidney) after the administration of the aforementioned treatment protocol (Fig. S14G). These results further demonstrated that Ab-MMB_1532_ possesses excellent biocompatibility *in vivo*.

## 4 Conclusion

In this study, we developed a dual-target drug-delivery system capable of site-specific drug accumulation and release within AS plaques. Specifically, Ab-MMB_1532_, equipped with CD86 antibody-mediated targeting and magnetic guidance, efficiently recognized M1 macrophages *in vitro*. Notably, ultrasound-responsive BIBR1532 released from the Ab-MMB_1532_ triggered M1 macrophage apoptosis by inhibiting the TERT/NF-κB signaling axis. In an AS mouse model, Ab-MMB_1532_ exhibited robust plaque-selective accumulation, and therapeutic outcomes demonstrated the effective attenuation of AS progression following treatment. In addition, prolonged administration of Ab-MMB_1532_ resulted in no observable adverse effects, confirming its favorable safety profile. Thus, this novel system with dual-target capabilities has the potential for use in the treatment of AS.

## CRediT authorship contribution statement

**Wei Zeng:** Writing – original draft, Visualization, Validation, Software, Resources, Investigation, Formal analysis, Data curation. **Zhengan Huang:** Visualization, Software, Resources, Data curation. **Yalan Huang:** Resources. **Kaifen Xiong:** Resources. **Yuanyuan Sheng:** Investigation. **Xiaoxuan Lin:** Investigation. **Xiaofang Zhong:** Resources. **Jiayu Ye:** Resources. **Yanbin Guo:** Resources. **Gulzira Arkin:** Resources. **Jinfeng Xu:** Supervision, Funding acquisition, Conceptualization. **Hongwen Fei:** Conceptualization. **Yingying Liu:** Writing – review & editing, Validation, Supervision, Project administration, Methodology, Funding acquisition, Conceptualization.

## Declaration of competing interest

The authors declare the following financial interests/personal relationships which may be considered as potential competing interests: Yingying Liu reports financial support was provided by 10.13039/501100001809National Natural Science Foundation of China. Yingying Liu reports financial support was provided by Project of Innovation of the Science and Technology Commission of Shenzhen City. Yingying Liu reports was provided by Project of International cooperative research of the Science and Technology Commission of Shenzhen City. If there are other authors, they declare that they have no known competing financial interests or personal relationships that could have appeared to influence the work reported in this paper.

## Data Availability

Data will be made available on request.
