# Supplementary material for: Dual-targeted microbubbles for atherosclerosis therapy: Inducing M1 macrophage apoptosis by inhibiting telomerase activity
Source: Mater Today Bio. 2025 Mar 20;32:101675. doi: 10.1016/j.mtbio.2025.101675 (PMC11986608; doi:10.1016/j.mtbio.2025.101675)
Supplement: Multimedia component 1 [file mmc1.docx]

**Dual-Targeted Microbubbles for Atherosclerosis Therapy: Inducing M1 Macrophage Apoptosis by Inhibiting Telomerase Activity**

Wei Zeng^1‡^, Zhengan Huang^2‡^, Yalan Huang^1,3^, Kaifen Xiong^4,5^, Yuanyuan Sheng^1^, Xiaoxuan Lin^1^, Xiaofang Zhong^1^, Jiayu Ye^1^, Yanbin Guo^1^, Gulzira Arkin^1^, Jinfeng Xu^1*^, Hongwen Fei^2*^, and Yingying Liu^1*^

**Materials and methods**

**1. Antibodies and reagents**

Antibodies against iNOS, CD86, [NF-κB p65, [Phospho-NF-κB p65](https://www.cellsignal.cn/products/primary-antibodies/phospho-nf-kb-p65-ser536-93h1-rabbit-mab/3033)](https://www.cellsignal.cn/products/primary-antibodies/nf-kb-p65-d14e12-xp-rabbit-mab/8242), caspase-3 and cleaved caspase-3 were purchased from Cell Signaling Technology (Boston, USA). Antibodies against β-actin, β-Tubulin, IκBα and Phospho-IκBα were purchased from Affinity Biosciences (California, USA). Antibodies against TERT and CD68 were purchased from Proteintech Group (Wuhan, China). Anti-rabbit HRP-conjugated IgG secondary antibody, lipopolysaccharide (LPS), Ammonium Bicarbonate (NH4HCO3), polyvinyl alcohol (PVA) was purchased from Sigma-Aldrich (St. Louis, USA). Dulbecco's modified Eagle's Medium (DMEM) and fetal bovine serum (FBS) were purchased from Invitrogen (CA, USA). 1,2-distearoyl-snglycero-3-phosphocholine (DSPC) were obtained from Avanti Polar Lipids (AL, USA). BIBR1532 were bought from MCE (New Jersey, USA). 100×penicillin-streptomycin solution, 1,1'-dioctadecyl-3,3',3'-tetramethylindocarbocyanine perchlorate (DiI), 2-(4-Amidinophenyl)-6-indolecarbamidine dihydro chloride (DAPI) and Senescence Cell Staining Kit were purchased from Beyotime Biotechnology (Shanghai, China). Poly (DL-lactide-co-glycolide) (PLGA, 50:50, MW = 30 000) were purchased from Jinan Daigang Biological Material (Shandong, China). Oleic acid-modified Fe_3_O_4_ nanoparticles were bought from Nanjing Nanoeast Biotech (Nanjing, China). Telomere Length Detection Kit were bought from [Biowing Applied Biotechnology](https://en.biowing.com.cn/) (Shanghai, China). Telomerase activity assay were bought from Key-GEN BioTECH (Jiangsu,China). Cell Counting Kit-8, Annexin V-PE / 7-AAD cell apoptosis detection kit and Protein Silver Stain Kit were purchased from Servicebio (Wuhan, China)

**2. Cell viability assay and Flow Cytometric (FC) analysis**

Cell viability was assessed using Cell Counting Kit-8. M1 macrophages were cultured with varying concentrations of BIBR1532 or microbubbles for 24 h. Following the kit instructions, cell viability was determined using the standard protocol. Experiments were conducted in triplicate.

Cell apoptosis was evaluated by FC. Briefly, following treatment of M1 macrophages with various concentrations of BIBR1532, cells were gently washed twice with pre-chilled PBS for a duration of 3 minutes each. Subsequently, the cells were harvested by cell scraping and centrifuged at 1000 rpm for 4 minutes at 4°C, and the supernatant was discarded. Cell staining was performed according to the instructions provided in the Annexin V-PE / 7-AAD cell apoptosis detection kit, with the entire procedure conducted in the absence of light and on ice. Cell apoptosis was assessed using a CytoFLEX flow cytometer (Beckman, USA) with FlowJo analysis software version 10.9.

**3. Quantitative Real-time PCR (qPCR)**

Following the manufacturer's protocol, total RNA was extracted from the cells using TRIzol^®^ reagent (Invitrogen, USA). The first-strand cDNA synthesis was per-formed using PrimeScipt^TM^ RT Master Mix (Takara, China). The cDNA and primers were mixed with TB Green^®^ Premix Ex Taq^TM^ II (Takara, China), and RT-qPCR was conducted on the StepOnePlus Real-Time PCR System (Thermo Fisher Scientific, USA) using the following thermal cycling program: 95°C for 30 seconds, followed by 40 cycles of 95°C for 5 seconds, 60°C for 30 seconds. β-actin was used as the endogenous reference gene, and relative gene expression was quantified using the 2^-ΔΔCq^ method^[1]^. The primers employed in this study were detailed in Table S2

**4. Western blot (WB)**

The cells, following the treatment above, were collected and subjected to cell lysis using RIPA buffer (Beyotime, China). Subsequently, the protein concentration was determined using a BCA assay kit (Sparkjade, China). Sodium dodecyl sulfate-polyacrylamide gel electrophoresis (SDS-PAGE) and WB were carried out in accordance with standard procedures.

**5. Immunofluorescence (IF) staining**

Frozen tissue sections or adherent cells were fixed with 4% paraformaldehyde (PFA, 10 min) followed by 0.1% Triton X-100 permeabilization (5 minutes). All specimens were blocked with 5% species-matched serum (1 hour) and incubated with primary antibodies (4℃/overnight) followed by fluorophore-conjugated secondary antibodies (RT/2 hours), with DAPI counterstaining (5 μg/ml, 5 minutes). Confocal imaging under standardized parameters was performed, and quantitative analysis was conducted via ImageJ software.

**6. Immunohistochemistry (IHC)**

Frozen arterial sections (8 μm) were fixed with cold PFA and endogenous peroxidase activity was blocked. After blocking non-specific binding, the slides were incubated overnight at 4°C with the primary antibody specific to the species. This was followed by incubation with an enzyme-labeled secondary antibody. Target proteins were visualized using DAB chromogen and counterstained with hematoxylin. Finally, the percentage of stained area was independently quantified using threshold analysis based on Image Pro Plus software (Version: 6.0).

**7. Telomerase activity assay and Protein Silver Stain Kit**

Telomerase activity was measured with the Fluorescent RT-qPCR assay kit. PCR products were separated by electrophoresis on a 10% nondenaturing polyacrylamide gel visualized by Protein Silver Stain Kit staining.

**8. Telomere length detection**

Telomere length was analyzed using SYBR Green-based qPCR. Genomic DNA was purified using magnetic beads, normalized to 10 ng/μL, and amplified in triplicate with telomere- and reference-specific primers. T/S ratios were calculated via four-point calibration curves derived from serially diluted mouse DNA standards, with technical replicates maintaining <0.3 Ct deviation.

**9. Senescence-associated β-galactosidase detection**

Cells were subjected to senescence-associated β-galactosidase (SA-β-Gal) staining using a Beyotime Senescence Cell Staining Kit. After a PBS washing, cells were fixed for 15 minutes with fixing solution, followed by overnight incubation in staining solution at 37°C. Senescent cells were identified by green staining.

**RESULTS**

**Table S1**. The particle sizes and PDI of MB_1532_ and MMB_1532_.

| Sample name | Type | Z-Ave (d.nm) | Mean ± SEM  (d.nm) | PdI | Mean ± SEM |
| --- | --- | --- | --- | --- | --- |
| MB_1532_ | Size | 1388 | 1398 ± 17.72 | 0.308 | 0.26 ± 0.07 |
|  | Size | 1418 |  | 0.164 |  |
|  | Size | 1379 |  | 0.312 |  |
|  | Size | 1407 |  | 0.246 |  |
| MMB_1532_ | Size | 2235 | 2291 ± 157.09 | 0.205 | 0.23 ± 0.10 |
|  | Size | 2094 |  | 0.270 |  |
|  | Size | 2404 |  | 0.105 |  |
|  | Size | 2430 |  | 0.331 |  |

**Table S2**. Primer sequences.

| Gene | Forward (5’-3’) | Reverse (5’-3’) |
| --- | --- | --- |
| iNOS | GCTGCCAGGGTCACAACTTTA | CAACGTTCTCCGTTCTCTTGCAG |
| TNF-α | GGTCCCCAAAGGGATGAGAAG | CACTTGGTGGTTTGTGAGTGTG |
| IL-1β | TGGCAACTGTTCCTG | GGAAGCAGCCCTTCATCTTT |
| TERT | GAAAGTAGAGGATTGCCACTGGC | CGTATGTGTCCATCAGCCAGAAC |
| β-actin | TATAAAACCCGGCGGCGCA | CAACGTTCTCCGTTCTCTTGCAG |

**Table S3**. Gene correlation analysis.

| gene | meta | coefficient | p-value | relation |
| --- | --- | --- | --- | --- |
| TERT | Lck | -0.378753279 | 0.354831819 | Negtive |
| TERT | Plau | -0.549480739 | 0.158319644 | Negtive |
| TERT | Lta | 0.593530389 | 0.120868959 | Positive |
| TERT | Tnf | 0.574943278 | 0.135988784 | Positive |
| TERT | Cd40 | 0.571122547 | 0.139220343 | Positive |
| TERT | Card11 | 0.41824689 | 0.302442746 | Positive |
| TERT | Eda2r | 0.669250025 | 0.069501917 | Positive |
| TERT | Bcl2a1a | 0.592446311 | 0.121723436 | Positive |
| TERT | Bcl2a1d | 0.580356882 | 0.131482133 | Positive |

| 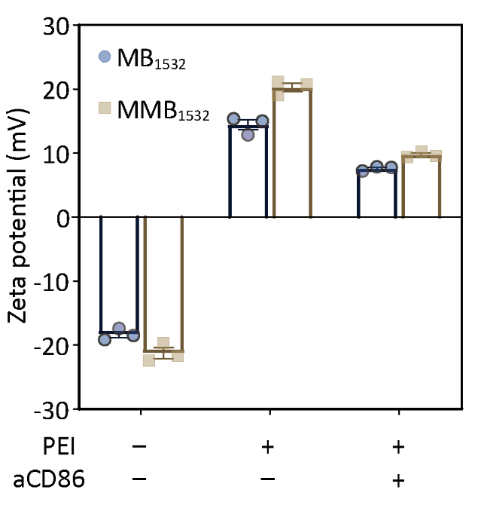 |
| --- |
| **Figure S1**. MB_1532_, MMB_1532_, and the zeta potentials modified with PEI and CD86 antibodies. |

| 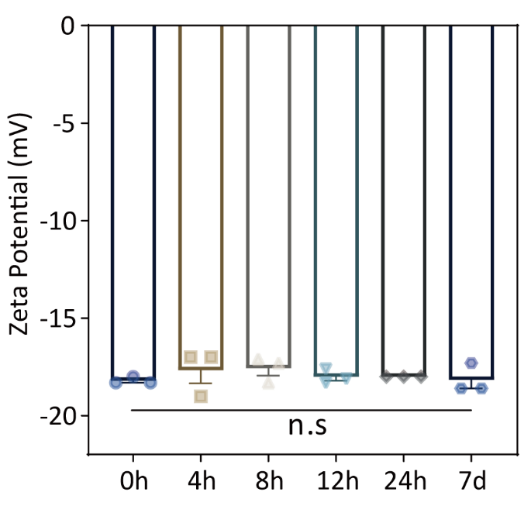 |
| --- |
| **Figure S2**. Stability of MB_1532_ measured by Malvern Zeta sizer. |

**The CD86 antibodies dissociation rate of Ab-MB_1532_ or Ab-MMB_1532_**

As described previously, Ab-MB_1532_ or Ab-MMB_1532_ was prepared via electrostatic adsorption using FITC Plus Anti-Mouse CD86 (Proteintech, FITC-65068). In this experiment, Ab-MMB_1532_ was used as an example:

| 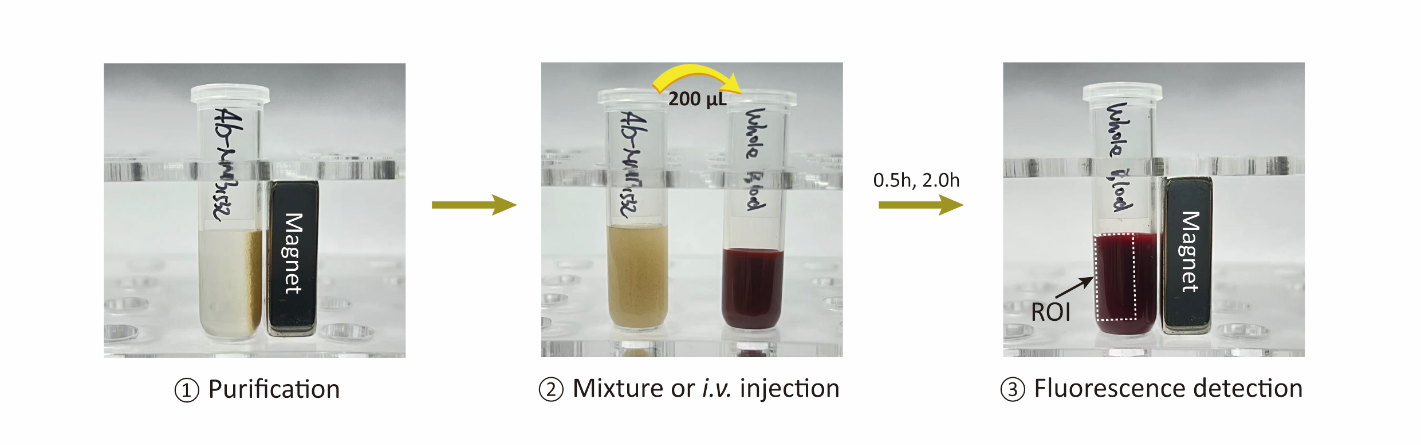 |
| --- |

First, a standard curve for FITC-CD86 antibody was constructed using a fluorescence spectrometer. Following this, Ab-MMB_1532_ was purified to remove unbound FITC-CD86 antibody or non-magnetic Ab-MMB_1532_. The purified Ab-MMB_1532_ was then mixed with whole blood or injected into the tail vein of mice. After 0.5 and 2 hours, fluid from the ROI and blood from the tail vein were collected for fluorescence detection. Then the dissociation rate of the FITC-CD86 antibody both in vitro and in vivo were calculated. All experiments were independently repeated 3 times to ensure the reliability of the results and were performed under light-protected conditions.

| 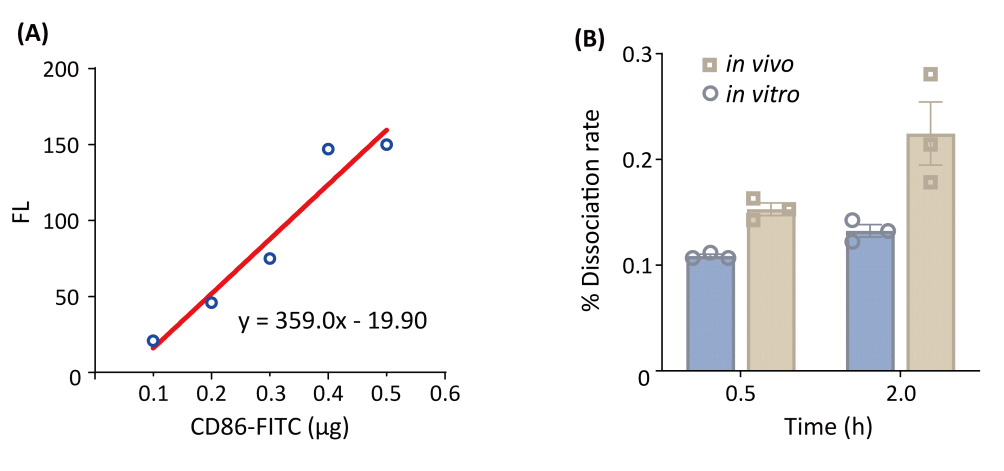 |
| --- |
| **Figure S3**. CD86 antibody dissociation rate on Ab-MMB_1532_. (A) The Concentration-Fluorescence Standard Curve of FITC-conjugated CD86 Antibody. (B) The dissociation rate of Ab-MMB_1532_ in vitro and in vivo within 2 hours. |

**The drug loading rate of MB_1532_ and MMB_1532_**

Firstly, a certain amount of BIBR1532 was dissolved in DMSO, then gradient dilution and full spectrum scanning (200-1000 nm) with UV/Vis spectrophotometer (TECAN, SPARK 10M, USA) were used to obtain the wavelength-absorbance (Wavle - OD) curve (Figure S3A). Then the absorbance values acquired at a given wavelength (here: 325nm) were used to calculate the absorption coefficient according to the Lambert-Beer’s law *A/L=αC*, where *A/L* is the absorbance per length, *α* is the extinction coefficient, and *C* is the concentration[2].

Then, the freeze-dried microbubbles were fully dissolved in DMSO, OD values were measured by UV/Vis spectrophotometer, and then corresponding concentrations were obtained according to the above formula, and finally the freeze-dried drug content was obtained.

| 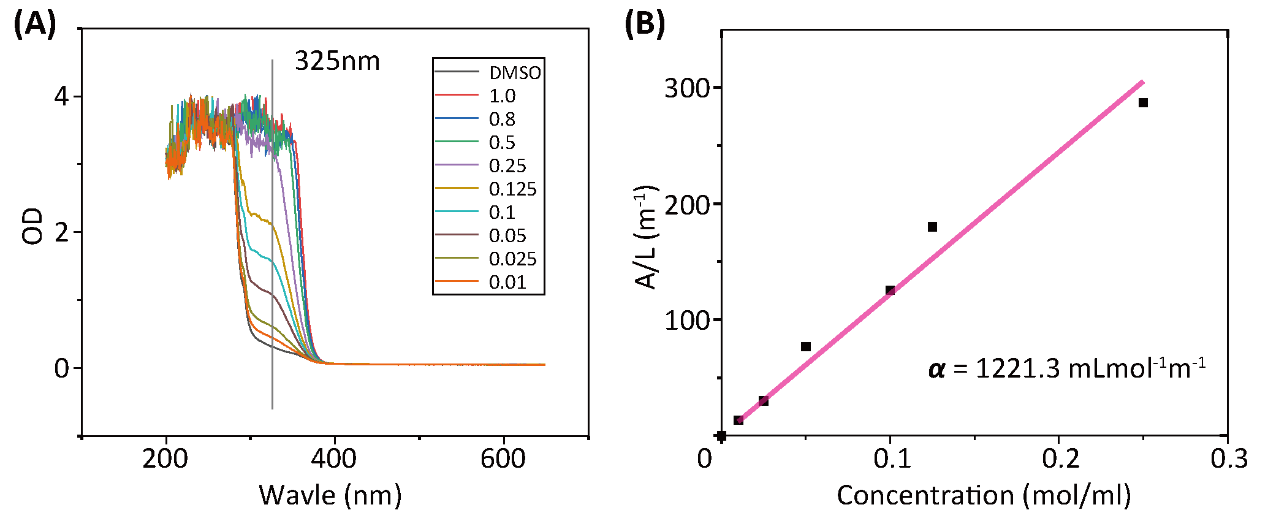 |
| --- |
| **Figure S4**. The drug loading rate of MB_1532_ and MMB_1532_. (**A**) Full spectrum scanning of BIBR1532 solution (solvent: DMSO). (B) Lamert-Beer plot of BIBR1532 suspension in DMSO. Each measurement was repeated three times. |

|  | |
| --- | --- |
| 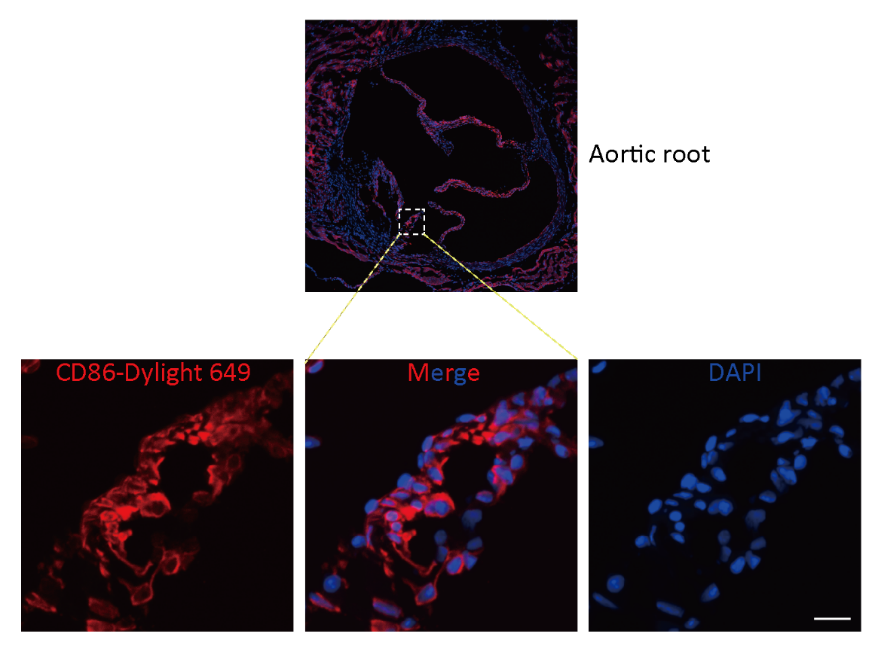 |  |
| **Figure S5**. Immunofluorescence staining was used to evaluate the expression of CD86 in atherosclerotic plaques. |  |

**Polarization and identification of M1 macrophages**

In order to evaluate the phenotypic change of macrophages, IF staining for the M1 macrophage (derived from RAW264.7 cells and BMDM) surface marker CD86 was conducted. As shown in Figure S6A & S7A, cells underwent a remarkable morphological transformation after treated by LPS for 24h. Macrophage in control exhibited with rounded and clustered appearance, while LPS group displayed with enlarged cell nuclei and elongated cell shapes, characterized by longer pseudopodia. The degree of cell elongation, which was defined as the longest axis length to the shortest axis length through cell nucleus, was quantified (Figure S6B) [2]. The cell elongation rate of LPS group was about 3 times that of the control group, as well as the average fluorescence intensity of CD86 was approximately 1.3 times that of the controls (*p* < 0.05), confirming that LPS stimulates morphological changes in macrophages, leading to a potential influence on their polarization status (Figure S6C, D). Moreover, the gene expression of M1 macrophages marker including iNOS, TNF-α, IL-1β and IL-6 (Figure S6E-G, S7B), as well as the specific proteins iNOS and CD86 (Figure S6H-J, S7C), were all remarkably higher than control (*p* < 0.05). In short, the above results indicate that LPS can effectively induce RAW264.7 cells or BMDMs to polarize towards the M1 phenotype.

| 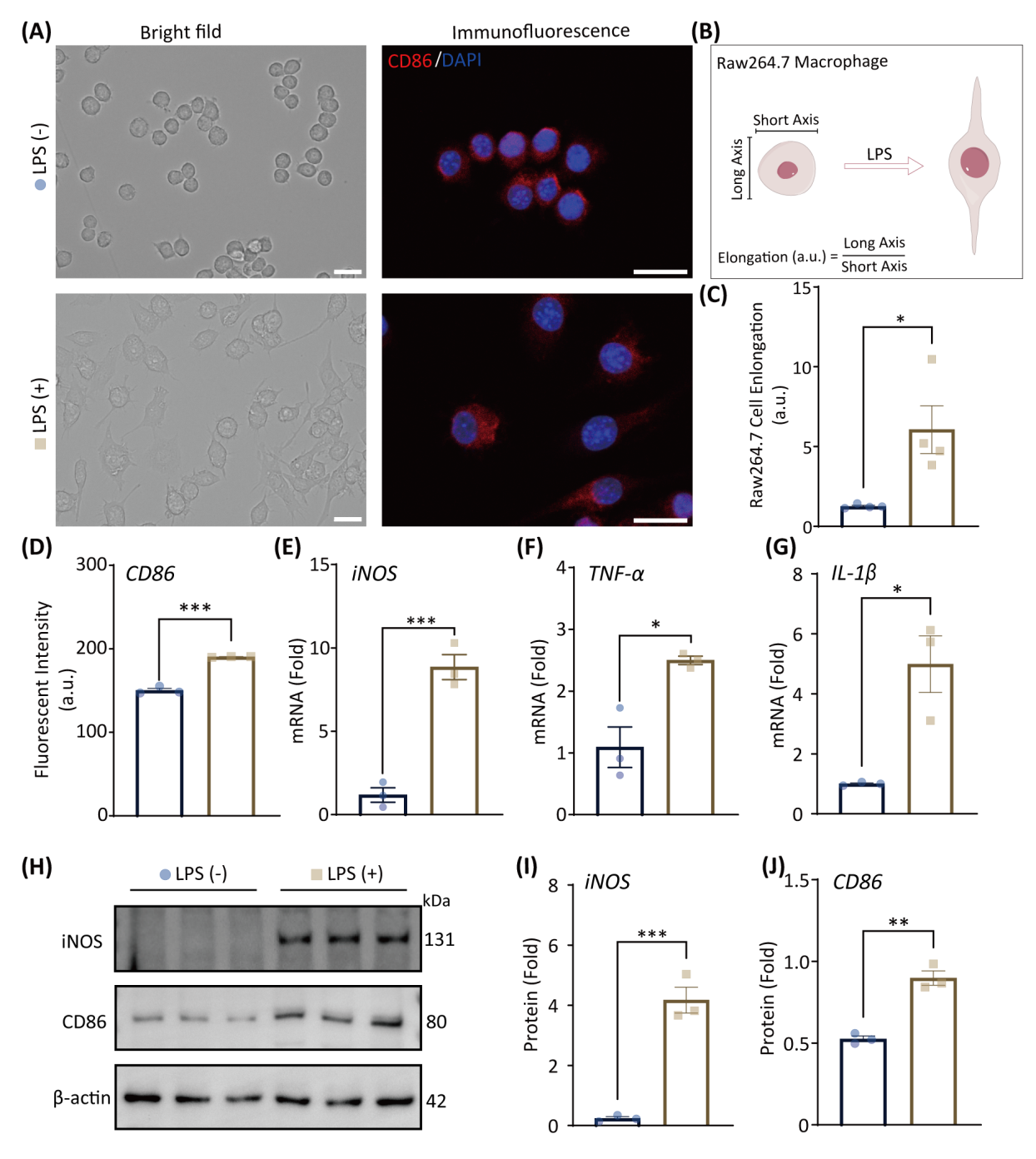 |
| --- |
| **Figure S6**. **Morphological, mRNA and protein expression changes of RAW264.7 macrophages following 24 h of LPS stimulation.** (**A**) Representative images of macrophage morphology and CD86 staining. Scale bar: 20 µm. (**B**) Schematic diagram and calculation formula of cell morphology changes, and (**C**) quantitative analysis. (**D**) Semiquantitative analysis of average fluorescence intensity of CD86. (**E-G**) The relative mRNA expression level of iNOS, TNF-α and IL-1β. (H-J) WB analysis of iNOS and CD86 protein levels. **p* < 0.05, ***p* < 0.01, and ****p* < 0.001. |

| 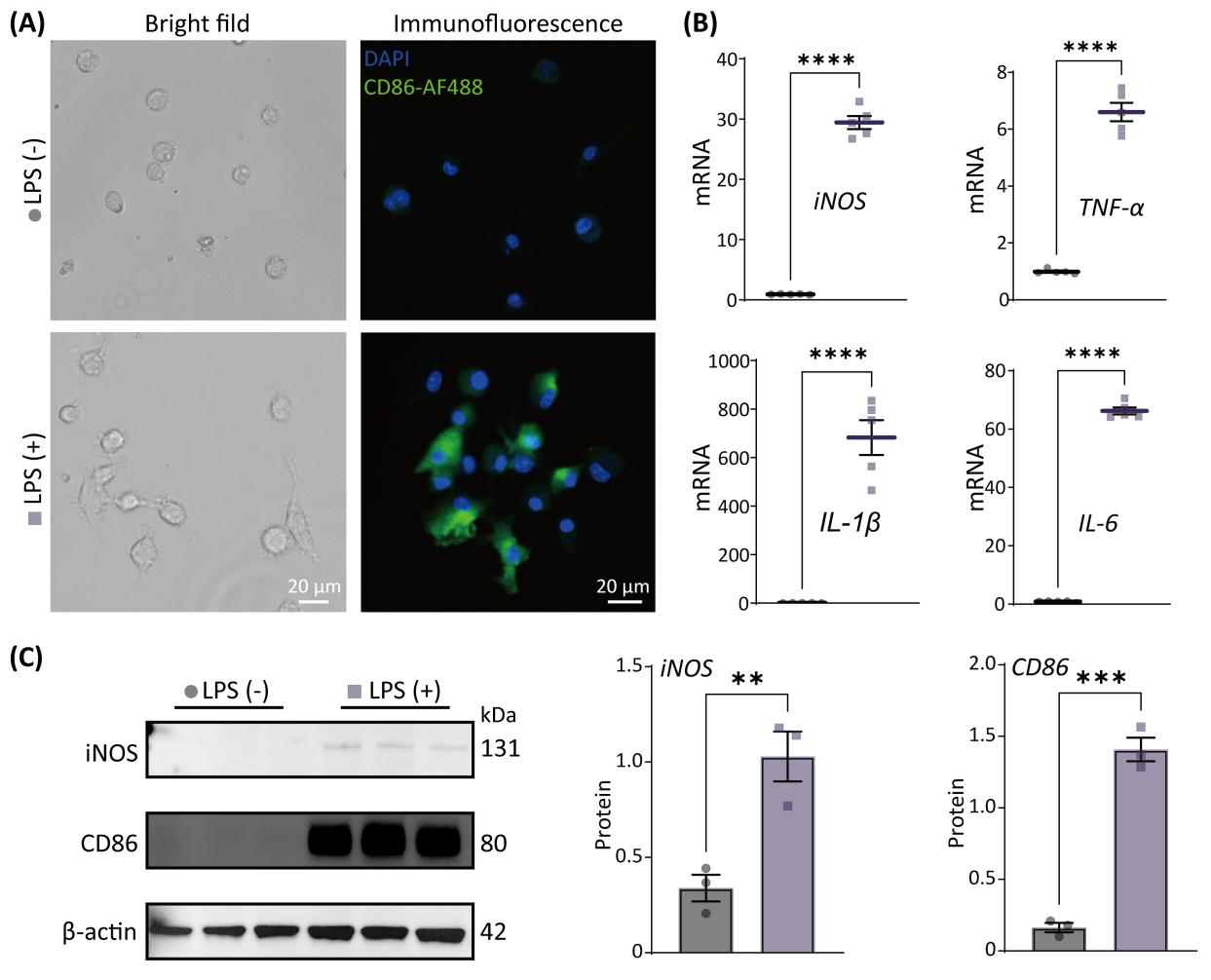 |
| --- |
| **Figure S7**. **Morphological, mRNA and protein expression changes of BMDM following 24 h of LPS stimulation.** (**A**) Representative images of macrophage morphology and CD86 staining. (**B**) The relative mRNA expression level of iNOS, TNF-α, IL-1β and IL-6. (C) WB analysis of iNOS and CD86 protein levels. **p* < 0.05, ***p* < 0.01, and ****p* < 0.001. |

| 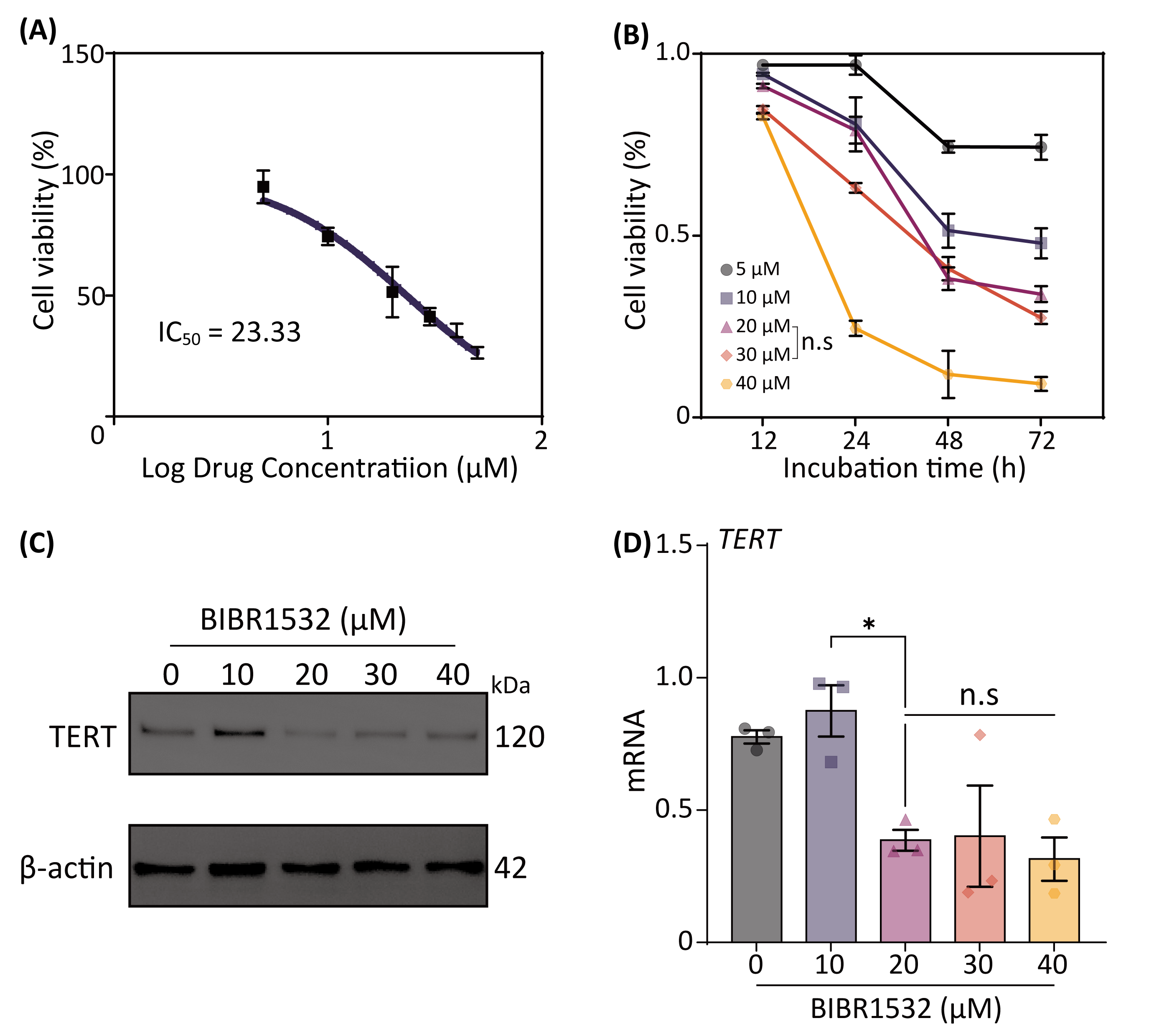 |
| --- |
| **Figure S8. Effect of BIBR1532 on M1 macrophages (BMDM derived). (A)** The IC_50_ value of BIBR1532 was evaluated using CCK8 assays. (**B**) Cell viability was evaluated by CCK8 assay at different time points. (**C**) The expression of TERT protein by WB. (**D**) The q-PCR analysis of TERT gene expression. |


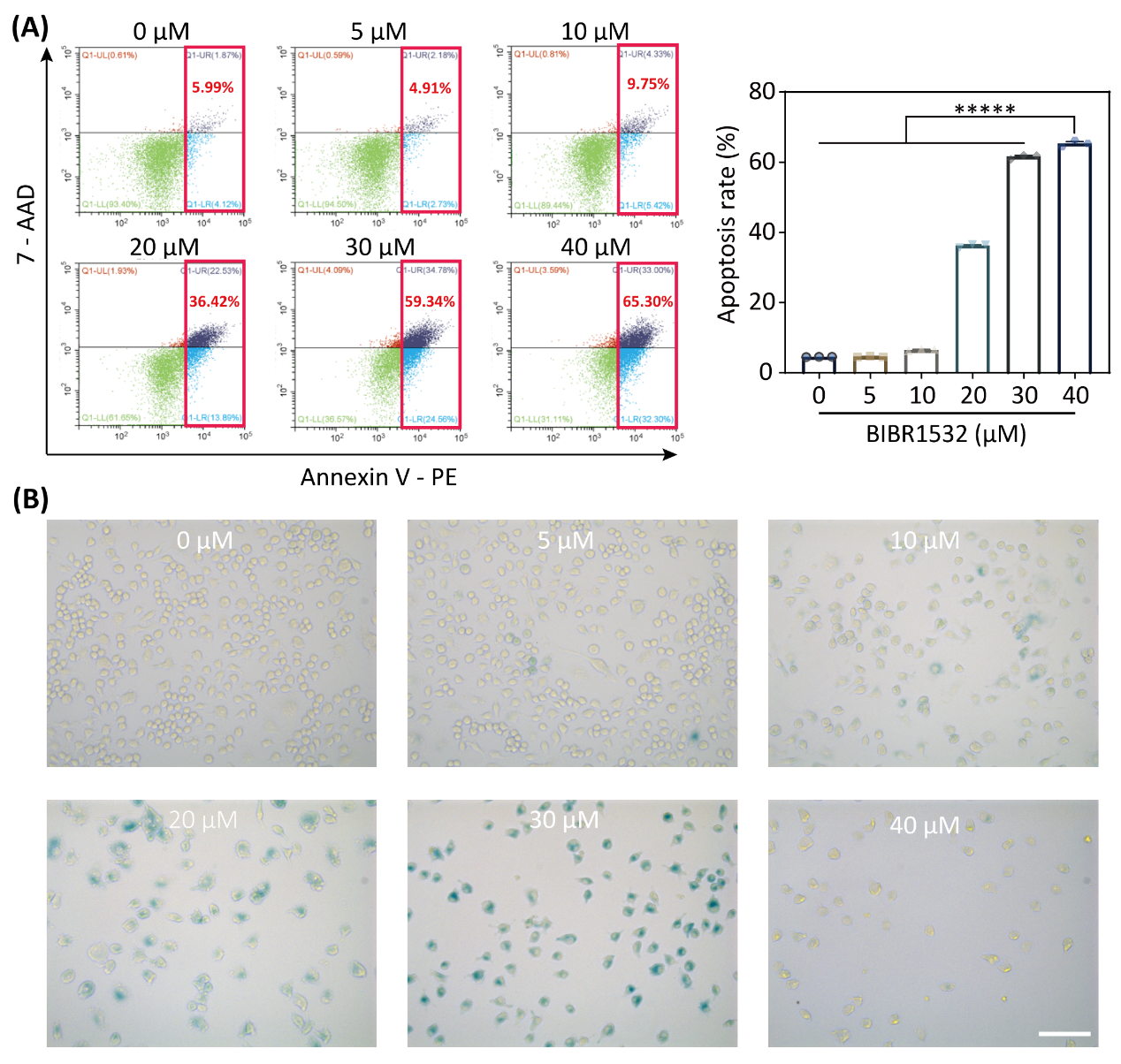


Figure S9. (A) Flow cytometry analysis of cell apoptosis and statistical graphs. (B) Cell senescence assessed with senescence-associated β-galactosidase staining. Scale bar: 20μM.

| 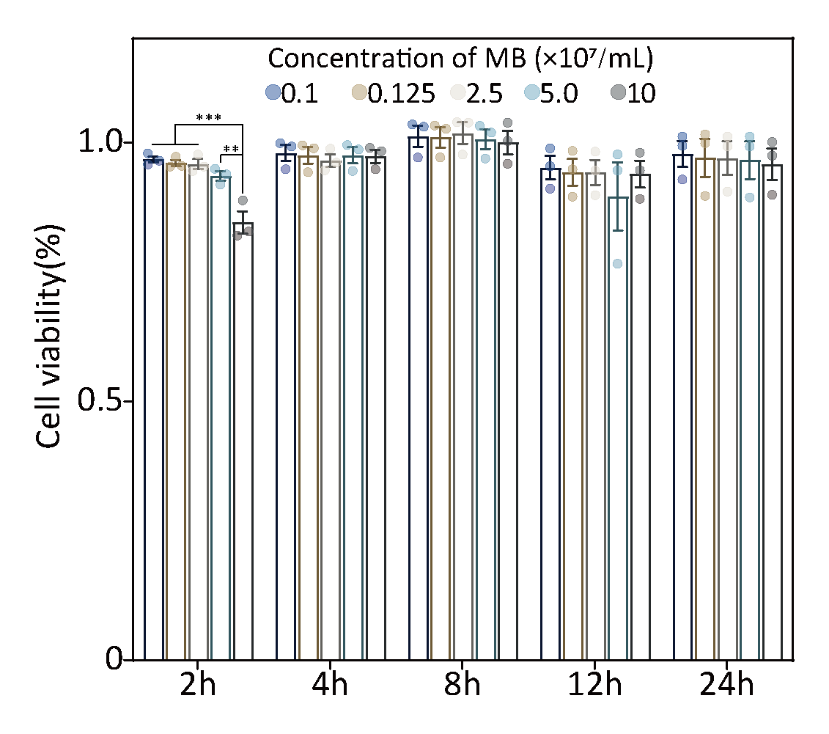 |
| --- |
| **Figure S10.** Effects of MB on M1 macrophages viability. |

| 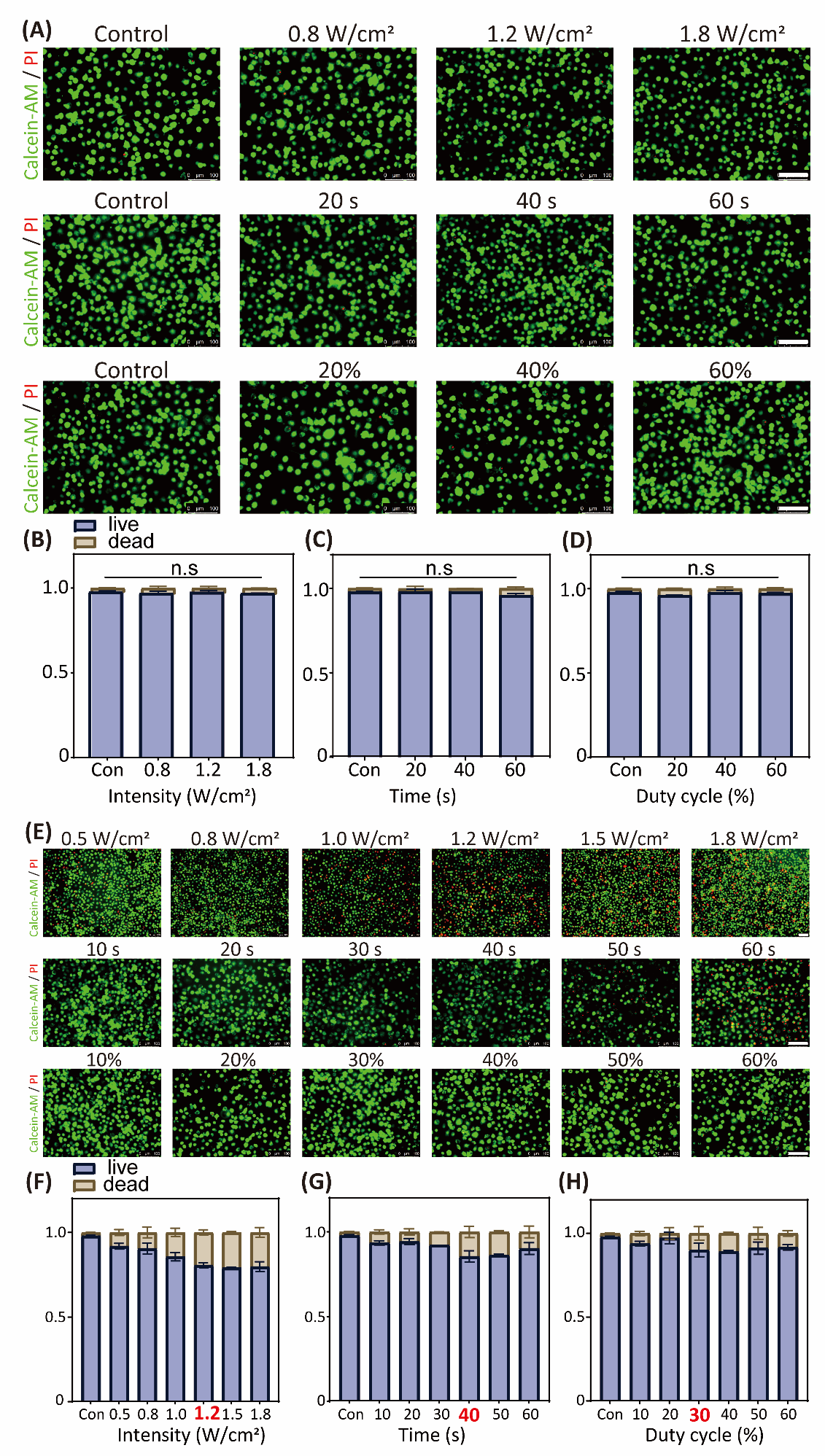 |
| --- |
| **Figure S11.** Study the ultrasound parameters for M1 macrophages. (A-D) The effect of ultrasound alone on cell apoptosis. (E-H) The effect of microbubbles on cell apoptosis under different ultrasound parameters, and the final parameter is 1.2 W/cm^2^ for intensity; 30% for duty cycle; 40 s for treatment time. |
| 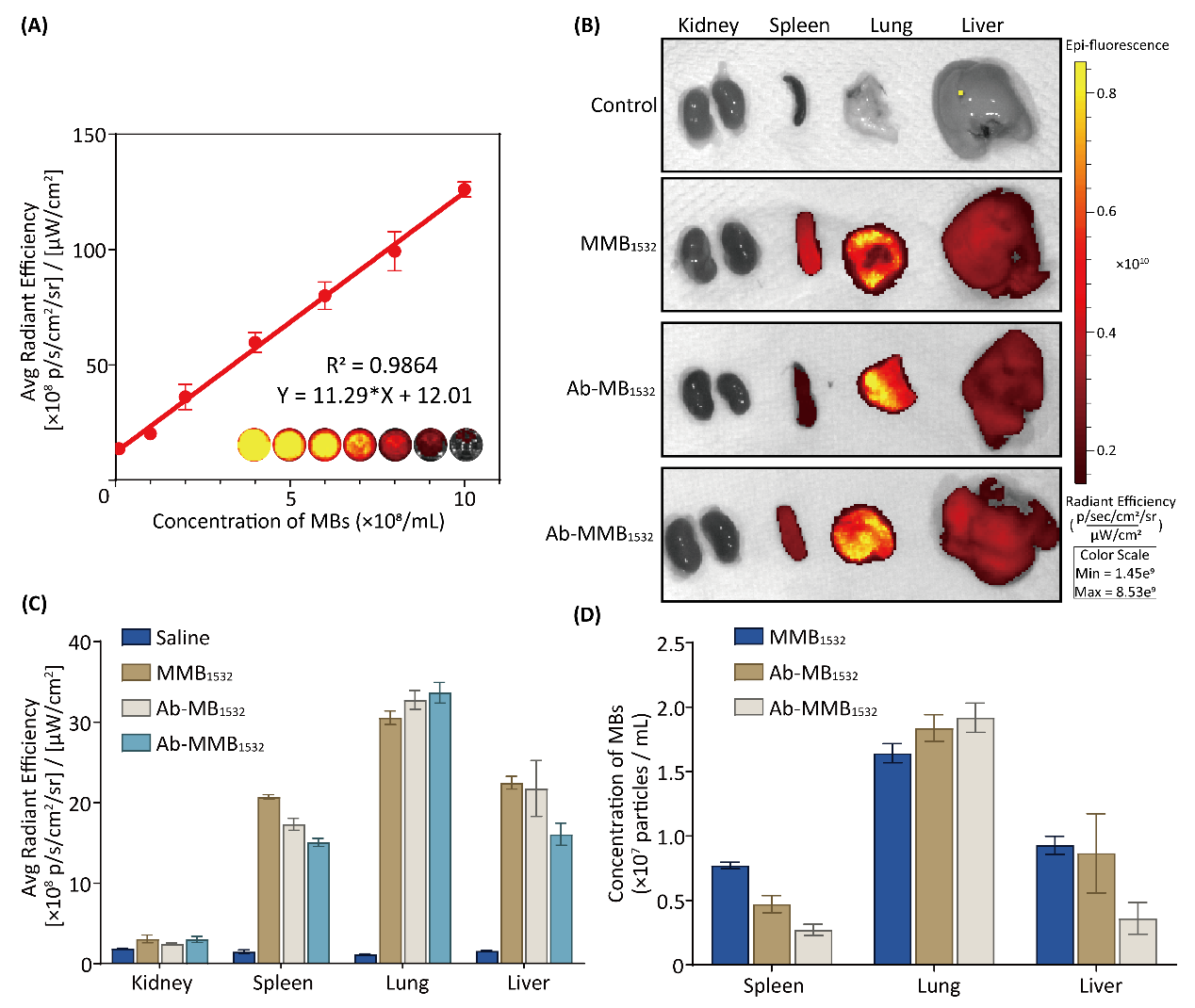 |
| **Figure S12.** The ***ex vivo*** fluorescent images of the main organs after the ***i.v.*** injection with DiD-labelled MMB_1532_, Ab-MB_1532_ and Ab-MMB_1532_ for 24h. |

| 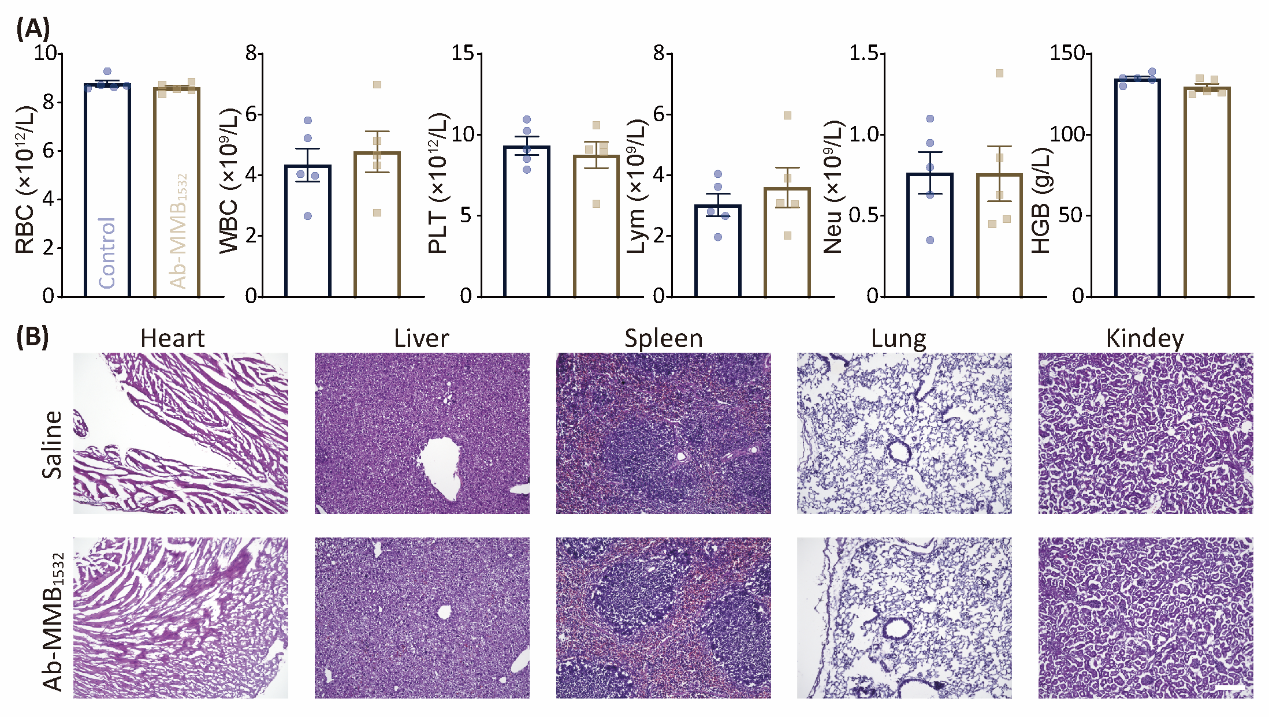 |
| --- |
| **Figure S13.** Acute toxicity of Ab-MMB_1532_ *in vivo*. (A) Typical hematological parameters (*n* = 5, mean ± SD). (B) HE stained of major organs. Scale bar: 200μM. |

| 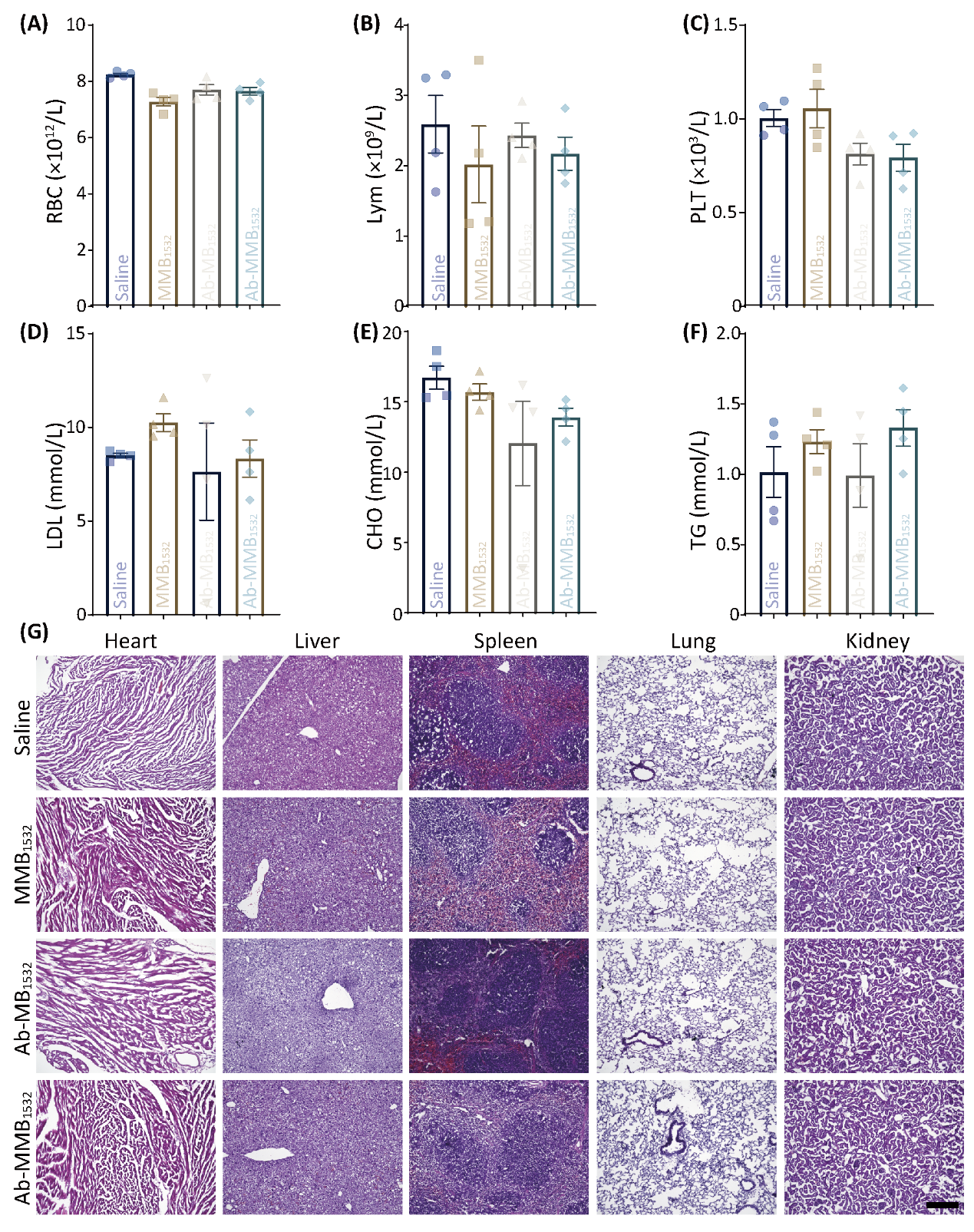 |
| --- |
| **Figure S14.** Safety assessment after treatment. (**A-C**) Hematological parameters, and (**D-F**) serum lipid level of each group of mice. (**G**) HE staining of major organs in each group of mice. Scale bar: 100 µm. |

**Reference**

[1] K.J. Livak, T.D. Schmittgen, Analysis of relative gene expression data using real-time quantitative PCR and the 2(-Delta Delta C(T)) Method, Methods (San Diego, Calif.) 25(4) (2001) 402-8.

[2] T. Zhang, Y. Wan, H. Xie, Y. Mu, P. Du, D. Wang, X. Wu, H. Ji, L. Wan, Degradation Chemistry and Stabilization of Exfoliated Few-Layer Black Phosphorus in Water, Journal of the American Chemical Society 140(24) (2018) 7561-7567.
