# Supplementary figures and images for: Dual-targeted microbubbles for atherosclerosis therapy: Inducing M1 macrophage apoptosis by inhibiting telomerase activity
Source: Mater Today Bio. 2025 Mar 20;32:101675. doi: 10.1016/j.mtbio.2025.101675 (PMC11986608; doi:10.1016/j.mtbio.2025.101675)

| 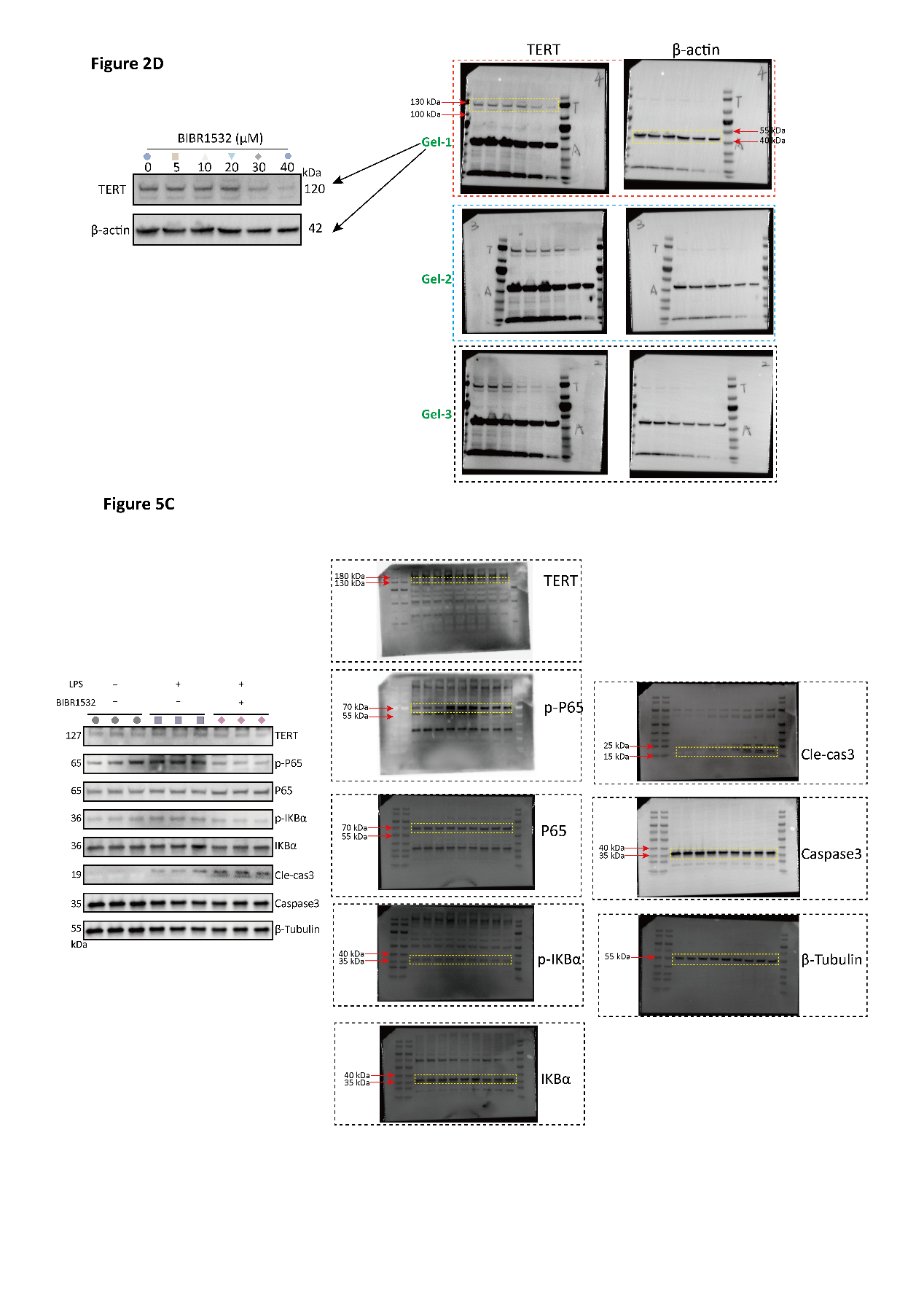 |
| --- |
| 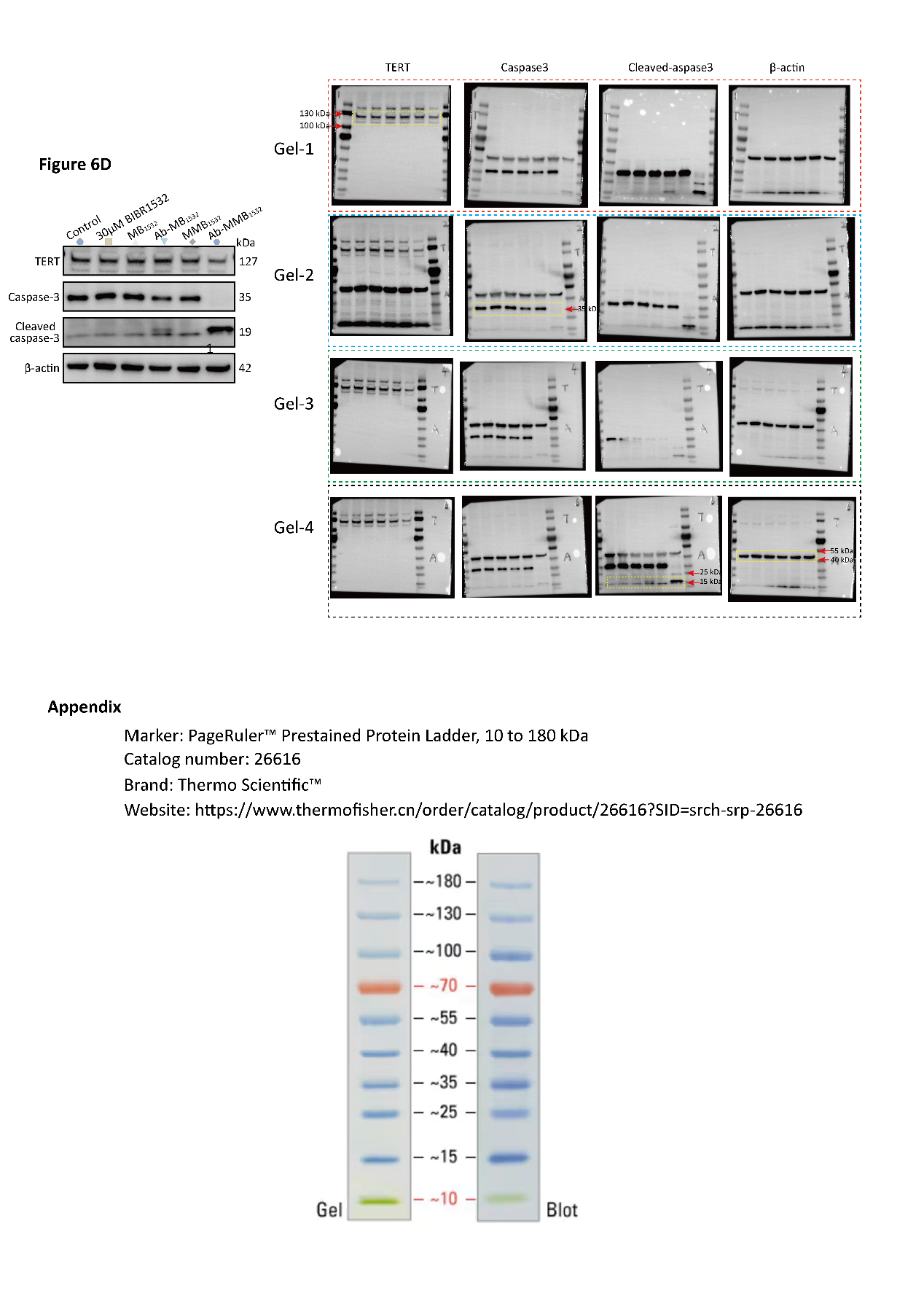 |
| 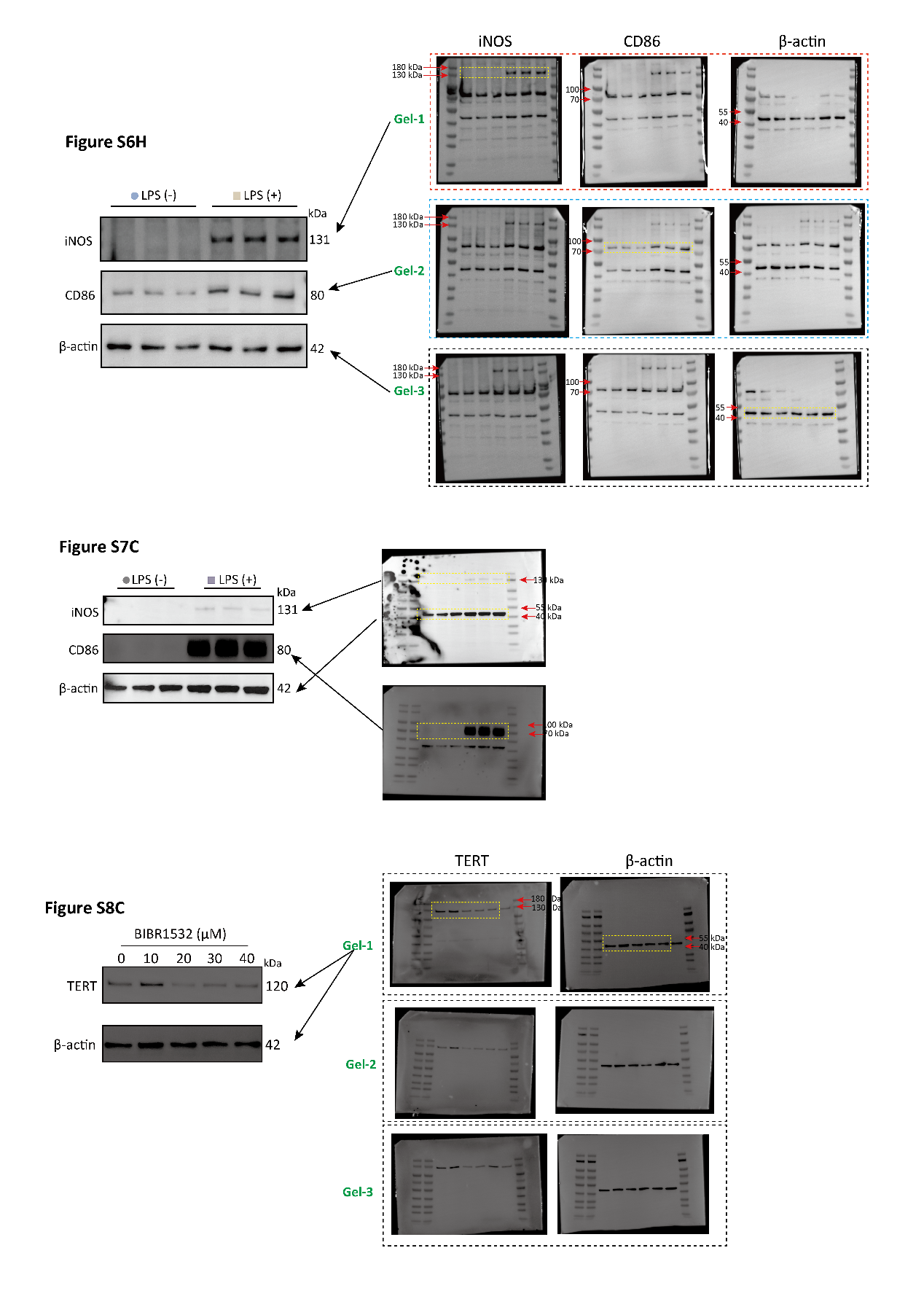 |

Supplement: Multimedia component 2 [file mmc2.docx]
